# Polysulfides promote protein disulfide bond formation in microorganisms growing under anaerobic conditions

**DOI:** 10.1128/aem.01926-24

**Published:** 2025-02-07

**Authors:** Yuping Xin, Qingda Wang, Jianming Yang, Xiaohua Wu, Yongzhen Xia, Luying Xun, Huaiwei Liu

**Affiliations:** 1 State Key Laboratory of Microbial Technology, Shandong University 12589 , Qingdao, China; 2 College of Life Sciences, Qingdao Agricultural University 98431 , Qingdao, China; 3 School of Molecular Biosciences, Washington State University 196198 , Pullman, Washington, USA; University of Tennessee at Knoxville, Knoxville, Tennessee, USA

**Keywords:** polysulfides, protein disulfide bond, anaerobic growth, DSB system, s-glutathionylation

## Abstract

Polysulfides commonly occur in anaerobic, microbial active environments, where they play key roles in sulfur cycling and redox transformations. Anaerobic survival of microorganisms requires the formation of protein disulfide bond (DSB). The relationship between polysulfides and anaerobic DSB formation has not been studied so far. Herein, we discovered that polysulfides can efficiently mediate protein DSB formation of microorganisms under anaerobic conditions. We used polysulfides to treat proteins, including roGFP2, Trx1, and DsbA, under anaerobic conditions and found that all three proteins formed intramolecular DSB *in vitro*. Under anaerobic conditions, *Escherichia coli* Δ*dsbB* displayed reduced growth and decreased intracellular protein DSB levels, but polysulfide treatment restored both growth and DSB content. Similarly, polysulfide treatment of *E. coli* Δ*dsbA* promoted periplasmic roGFP2 DSB formation and recovered growth under anaerobic conditions. Furthermore, treating *Schizosaccharomyces pombe* and *Cupriavidus pinatubonensis* JMP134 with polysulfides increased their intracellular protein DSB content. Collectively, these findings demonstrate that polysulfides can promote DSB formation independently of known enzymatic DSB-mediated systems and the presence of oxygen, thereby benefiting the survival of microorganisms in anaerobic habitats.

**IMPORTANCE:**

How polysulfides enhance the adaption of microorganisms to anaerobic environments remains unclear. Our study reveals that polysulfides efficiently facilitate protein DSB formation under anaerobic conditions. Polysulfides contain zero-valent sulfur atoms (S^0^), which can be transferred to the thiol group of cysteine residue. This S^0^ atom then accepts two electrons from two cysteine residues and is reduced to H_2_S, leaving the two cysteines linked by a disulfide bond. Anaerobic growth of microorganisms benefits from the formation of DSB. These findings pave the way for a deeper understanding of the intricate relationship between polysulfides and microorganisms in various environmental contexts.

## INTRODUCTION

Life on earth originated in an era predating the oxygen-rich atmosphere, where primitive cells relied on sulfur respiration for energy conversion. In this process, hydrogen sulfide was oxidized to sulfane sulfur (S^0^) (1, 2). It is plausible to hypothesize that S^0^ played significant roles in the physiology of these early cells, and some of these roles may persist in contemporary organisms. Polysulfides, which are low-molecular-weight thiol compounds containing S^0^, include octasulfur (S_8_), hydrogen polysulfide (HS_n_H, *n* ≥ 2), and organic polysulfide (RS_n_H, *n* ≥ 2 and RS_n_R, *n* ≥ 3). Polysulfides are commonly present in anaerobic habitats where microbial metabolisms are active, such as coastal sediments, in which their concentrations are up to 300–400 µM (3). They are mainly produced through the sulfate reduction pathway and the organic sulfur (such as cysteine and methionine) degradation pathway by microorganisms. In the past two decades, polysulfides have been intensively studied, and increasing evidence indicates that they are involved in a multitude of physiological processes in microorganisms, such as maintaining redox balance, regulating sulfur metabolism, and counteracting electrophilic stress (4–6).

Disulfide bonds (DSBs) are covalent linkages that connect the sulfur atoms of two cysteine residues within a protein. The formation of DSBs is essential for both aerobic and anaerobic growth of microorganisms (7, 8). In the periplasm and endoplasmic reticulum, DSB formation is generally considered to be an enzyme-catalyzed process. Since the discovery of the first DSB-forming enzymes in 1963 (9), several key players have been identified. For example, DsbA is recognized as a pivotal enzyme in the formation of DSBs in the bacterial periplasm (10, 11). In eukaryotic cells, the endoplasmic reticulum relies on protein disulfide isomerase (PDI) (12), while oxidase Mia40 plays a central role in mitochondria (13, 14). Under aerobic conditions, these enzymatic systems use oxygen (O_2_) as the final electron acceptor. Whereas under anaerobic conditions, specific electron acceptors are used. For instance, the DsbAB system uses fumarate or nitrate reductase, and the PDI-Ero1 system uses flavin adenine dinucleotide (FAD) as the final electron acceptors under anaerobic conditions (15–18). While these enzymatic systems are undoubtedly crucial for the formation of DSBs, they may not be absolutely essential (19, 20). It is likely that additional, yet-to-be-unveiled enzymes or factors are involved in the DSB formation process of the periplasm and endoplasmic reticulum.

DSBs also form within the cytoplasm. This process is typically facilitated by low-molecular-weight chemical agents, such as reactive oxygen species (ROS), reactive nitrogen species (RNS), and oxidized glutathione (GSSG) (21–24). Enzymes with redox activities, such as glutaredoxin and thioredoxin, also facilitate this process. It is noteworthy that these same agents—ROS, RNS, and GSSG—also trigger S-glutathionylation (Pr-SSG), which is the formation of a mixed disulfide bond between glutathione (GSH) and a protein (25–27). Both cytoplasmic DSBs and S-glutathionylation can take place under anaerobic conditions, and the specific factors that mediate their formation remain to be fully elucidated.

In this study, we first performed *in vitro* experiments and found that polysulfides can promote the formation of DSBs in purified proteins under anaerobic conditions. Second, we performed *in vivo* experiments and observed that polysulfides can promote DSB formation in anaerobically cultivated microorganisms. Our study revealed that polysulfides are beneficial for the survival of microorganisms in anaerobic habitats by promoting DSB formation independently of enzymatic systems.

## RESULTS

### Octasulfur-mediated GSH oxidation under anaerobic conditions

**Fig 1:**
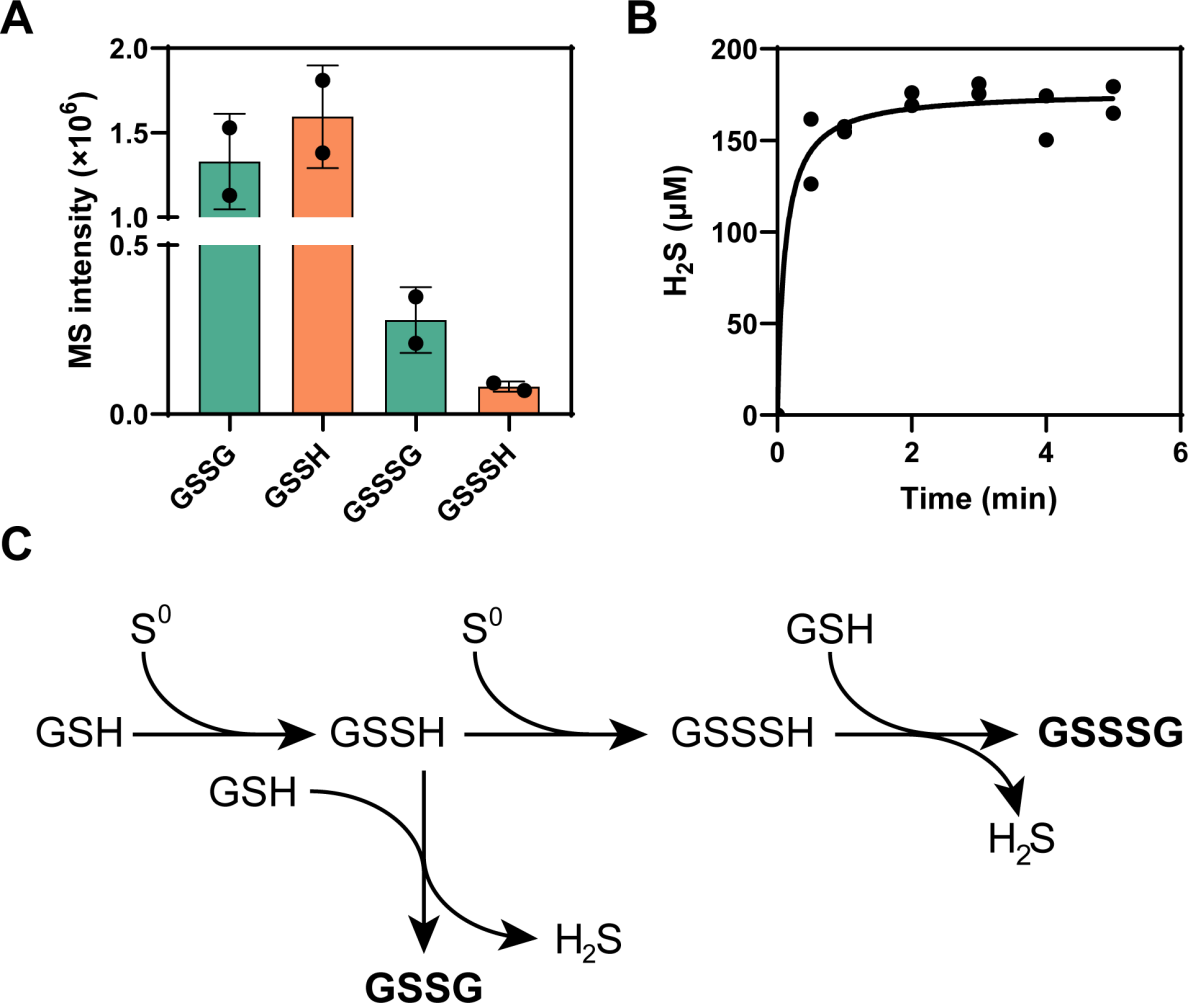
Reactions of GSH and S_8_ under anaerobic conditions. (**A**) The MS signal intensities of GSSG, GSSH, GSSSG, and GSSSH products. (**B**) Production of H_2_S in different reaction times. (**C**) The serial reactions between GSH and S_8_.

The small peptide GSH is prone to be oxidized into GSSG. To investigate the potential of polysulfides in mediating GSH oxidation,(Fig. 1) we mixed GSH with S_8_ (5 mM of each) in potassium phosphate (KPI) buffer (100 mM, pH 7.4) under anaerobic conditions. After 30 min incubation at room temperature, the resulting products were analyzed using liquid chromatography–electrospray ionization mass spectrometry (LC–ESI-MS). GSSG and GSSH (glutathione persulfide) were the predominant products, with MS signal intensities of 1.33 × 10^6^ and 1.59 × 10^6^, respectively (Fig. 1A; Fig. S1A and B). Additionally, GSSSH and GSSSG, which are secondary reaction products, were detected with comparatively lower MS signal intensities of 8.2 × 10^4^ and 2.78 × 10^5^, respectively (Fig. 1A; Fig. S1C and D). The reaction between GSH and S_8_ was rapid. H_2_S production was detectable within 1 min after mixing and reached a plateau within 3 min (Fig. 1B). No H_2_S, GSSG, GSSH, or other derivatives were detected from GSH solution without reacting with S_8_.

These findings suggest that polysulfides can catalyze the oxidation of GSH under anaerobic conditions through a series of reactions (Fig. 1C). The presence of GSSH implies that S^0^ initially reacts with one GSH molecule to form GSSH. This intermediate then reacts with another GSH molecule, resulting in the formation of GSSG and H_2_S. GSSSH and GSSSG are products generated by further reactions of GSSH with S^0^ and GSH.

### Hydrogen persulfide mediated DSB formation under anaerobic conditions *in vitro*

Three proteins capable of forming intramolecular DSBs were subjected to a reaction with hydrogen persulfide (HSSH) under anaerobic conditions. These proteins were expressed in and purified from *E. coli* BL21(DE3), each containing an N-terminal His Tag (Fig. S2). The purified proteins were dissolved in DTT containing buffer to keep their cysteine residues reduced. The protein–HSSH reactions were conducted in KPI buffer (10 mM, pH 7.4, DTT-free) at room temperature for 30 min under anaerobic conditions.

**Fig 2:**
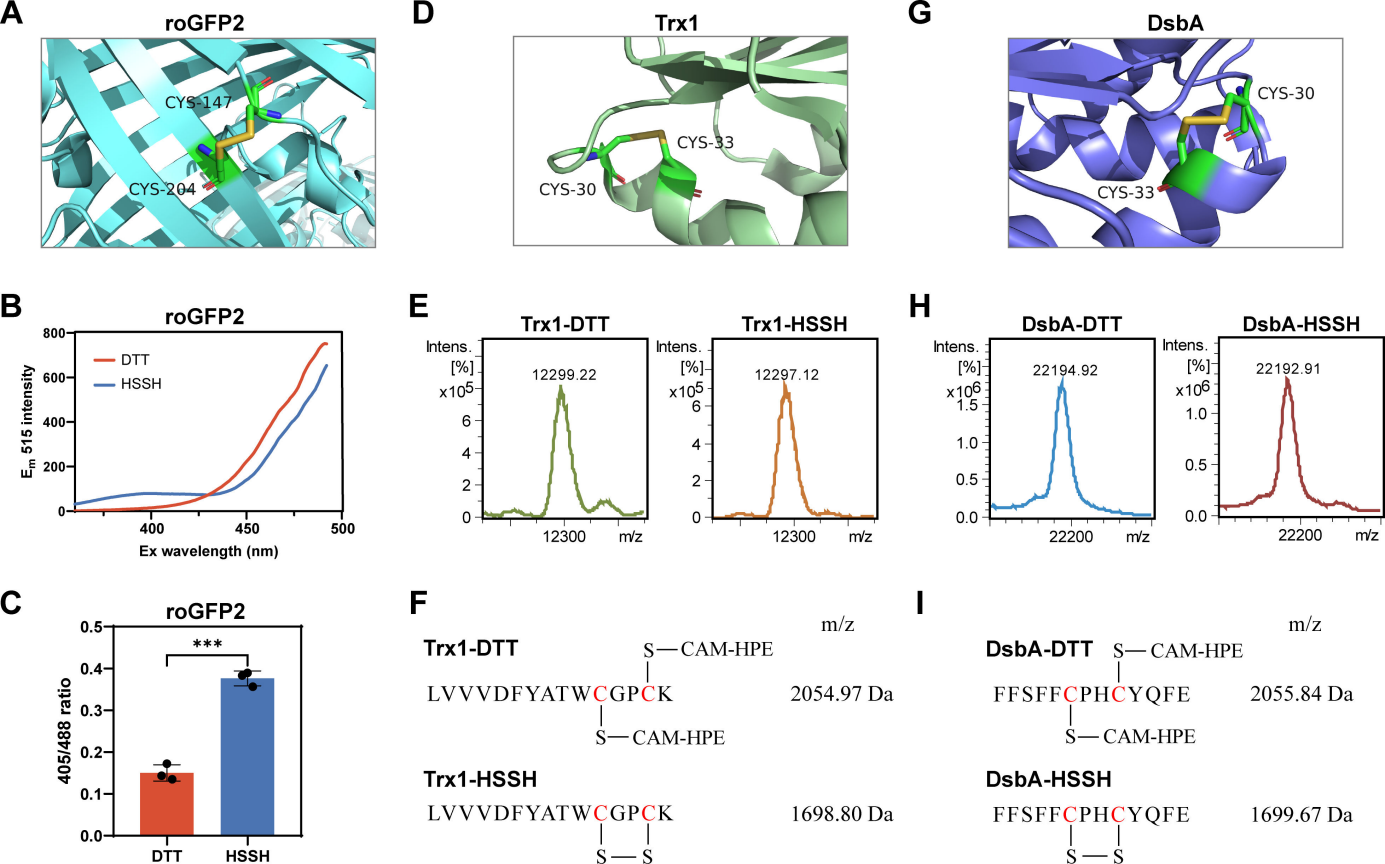
Proteins formed disulfide bonds by reacting with HSSH under anaerobic conditions. (**A**) 3D structure of roGFP2 with a disulfide bond between Cys_147_ and Cys_204_ (PDB ID, 1JC1). (**B**) Full excitation spectra of roGFP2. After HSSH treatment, the excitation peak of roGFP2 around 405 nm was increased, and the excitation peak around 488 nm was decreased. (**C**) HSSH-treated roGFP2 showed a higher ratio of 405/488 than did the untreated one. (**D**) 3D structure of Trx1 with a disulfide bond between Cys_30_ and Cys_33_ (PDB ID, 3F3Q). (**E**) Full mass spectra of Trx1 and HSSH-reacted Trx1. (**F**) Identified peptides from untreated or HSSH-reacted Trx1. (**G**) 3D structure of DsbA with a disulfide bond between Cys_30_ and Cys_33_ (PDB ID, 1A2M). (**H**) Full protein mass spectra of DsbA and HSSH-reacted DsbA. (**I**) Identified peptides from untreated or HSSH-reacted DsbA. For (C), data were from three independent repeats and presented as mean value ±s.d. *** represents *P* < 0.001 based on a two-sided *t*-test.

The first protein examined was roGFP2, a widely utilized redox-sensitive fluorescent protein derived from GFP. The roGFP2 features a mutation that allows its excitation spectrum to shift upon the formation of a DSB between Cys_147_ and Cys_204_ (Fig. 2), which are in close proximity to its chromophore (Fig. 2A). The formation of this DSB confers a new excitation wavelength (*E_x_* = 405 nm) to roGFP2 and reduces the efficacy of the original excitation wavelength (*E_x_* = 488 nm). Consequently, the ratio of emission light intensity excited at 405 nm to that excited at 488 nm (405/488) is proportional to the ratio of oxidized to reduced roGFP2 (roGFP_ox_/roGFP_re_) (28, 29).

Upon examining the excitation spectrum of HSSH-reacted roGFP2, a distinct new excitation peak emerged around 405 nm, while the excitation efficacy around 488 nm was diminished (Fig. 2B). In contrast, the control, roGFP2 in DTT containing KPI buffer (denoted as DTT-reacted roGFP2), showed only the excitation peak around 488 nm. Furthermore, the 405/488 ratio for HSSH-reacted roGFP2 was significantly higher than that of the DTT-reacted roGFP2 (Fig. 2C). These observations confirmed the formation of an intramolecular DSB in roGFP2 following its reaction with HSSH.

The second protein, thioredoxin 1 (Trx1) from *Saccharomyces cerevisiae*, is a cytoplasmic protein that participates in various redox reactions through its reversible DSB formed between Cys_30_ and Cys_33_ (Fig. 2D). Both HSSH-reacted and DTT-reacted Trx1 were analyzed using LC–MS and LC-MS/MS. At the whole protein level (analyzed by LC–MS), the molecular weight of the former was 12,297, which was two less than that of the latter (1) (Fig. 2E). At the peptide level (analyzed by LC–MS/MS), a peptide containing Cys_30_ and Cys_33_ was identified in the DTT-reacted Trx1, with both cysteines in the reduced form (–SH) and blocked by β-(4-hydroxyphenyl)ethyl iodoacetamide (HPE-IAM) (Fig. 2F; Fig. S3). The same peptide was also identified in HSSH-reacted Trx1 but with a key difference: the cysteines formed a DSB that was not blocked by HPE-IAM (Fig. 2F; Fig. S4). These analyses confirmed the formation of a DSB in HSSH-reacted Trx1 under anaerobic conditions.

The third protein, *E. coli* DsbA, is the primary facilitator of disulfide bond formation in secreted proteins. It functions by transferring its intra DSB, which is formed between Cys_30_–X–X–Cys_33_ (excluding the signal peptide), to substrate proteins (Fig. 2G). Both HSSH-reacted and DTT-reacted DsbA were subjected to LC–MS and LC–MS/MS analysis. At the whole protein MS level, the former exhibited a molecular weight of 22,192, which was two lower than that of the latter (22,194) (Fig. 2H). At the peptide MS level, a peptide containing Cys_30_ and Cys_33_ was detected in DTT-reacted DsbA, with a molecular weight of 2,055.8 (Fig. 2I; Fig. S5), corresponding to the reduced form of these two cysteines (directly blocked by HPE-IAM). The same peptide was also detected in HSSH-reacted DsbA, but with a molecular weight of 1,699.6 (Fig. 2I; Fig. S6 ), indicating the formation of a DSB between the two cysteine residues. Collectively, these experiments demonstrated that polysulfides can mediate the formation of protein DSBs under anaerobic conditions.

### Hydrogen persulfide was as effective as H_2_O_2_ in mediating DSB formation *in vitro*

To investigate the capacity of HSSH to induce DSB formation in the presence of oxygen, we conducted an experiment using 10 µM roGFP2 in various solutions: a solution containing DTT at 200 µM, one with HSSH at 200 µM, one with S_8_ at 200 µM, one with diamide at 200 µM, one with H_2_O_2_ at 200 µM, and a normoxic solution with dissolved oxygen at approximately 250 µM. The incubation was carried out under aerobic conditions at room temperature for 30 min.

**Fig 3:**
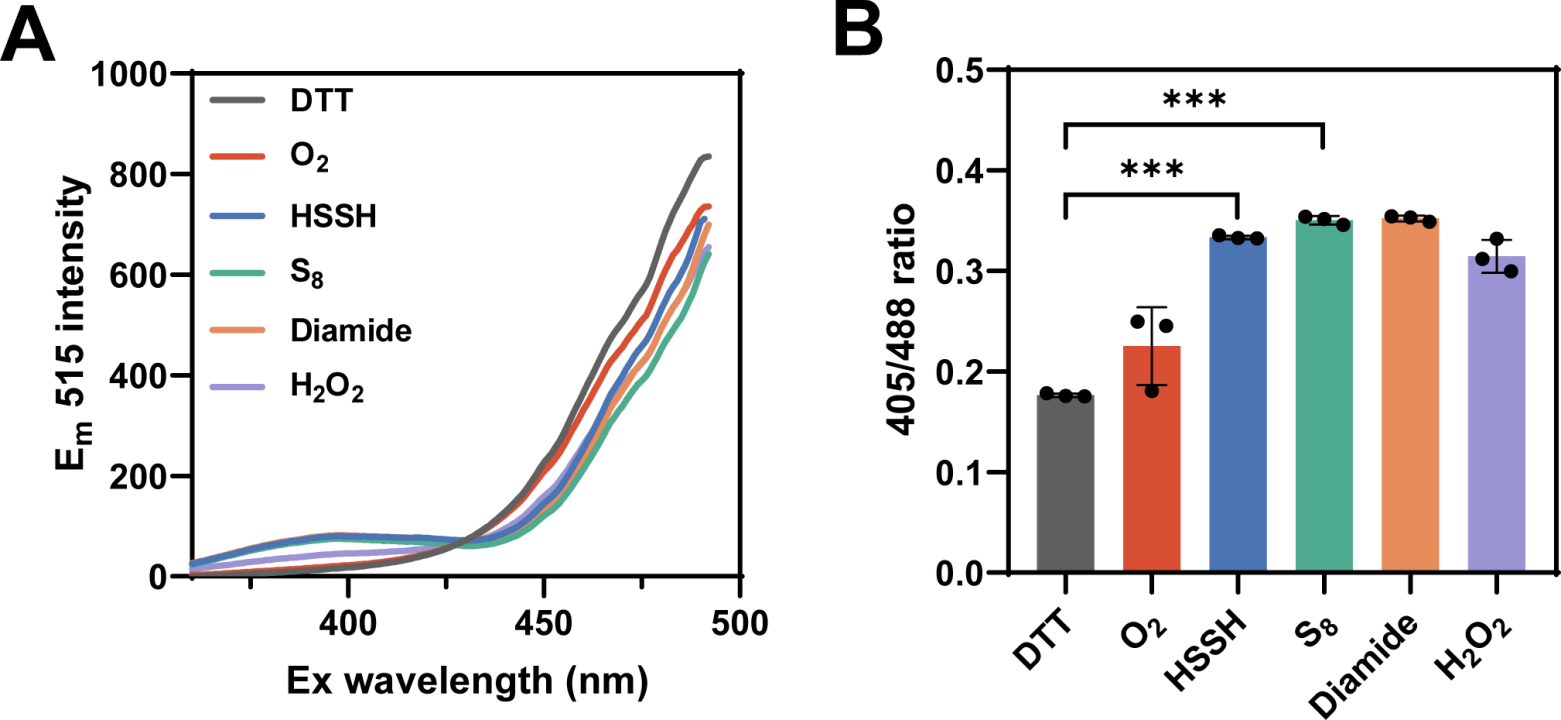
HSSH mediated roGFP2 DSB formation under aerobic conditions. (**A**) Full excitation spectra of roGFP2; 10 µM roGFP2 was reacted with 200 µM DTT, ~250 µM O_2_, 200 µM HSSH, 200 µM S_8_, 200 µM diamide, or 200 µM H_2_O_2_ under aerobic conditions. (**B**) The 405/488 ratios of roGFP2. Data were from three independent repeats and presented as mean value ±s.d. *** represents *P* < 0.001 based on a two-sided *t*-test.

Analysis of the excitation spectrum revealed distinct characteristics for each solution: roGFP2 treated with S_8_, HSSH, and diamide all exhibited a pronounced peak at 405 nm, roGFP2 treated with H_2_O_2_ showed a moderate peak; roGFP2 exposed to O_2_ had a weaker peak, and roGFP2 treated with DTT displayed no peak at 405 nm (Fig. 3A). The ratio of 405 nm to 488 nm excitation for HSSH and S_8_ -treated roGFP2 was significantly higher than that for DTT and O_2_ treated samples, and comparable to that for diamide and H_2_O_2_ treated ones (Fig. 3B). These findings suggest that HSSH and S_8_ were more effective than O_2_ and as efficient as diamide and H_2_O_2_ in facilitating the formation of DSB.

### Octasulfur compensated for *dsbB* deletion in *E. coli*

**Fig 4:**
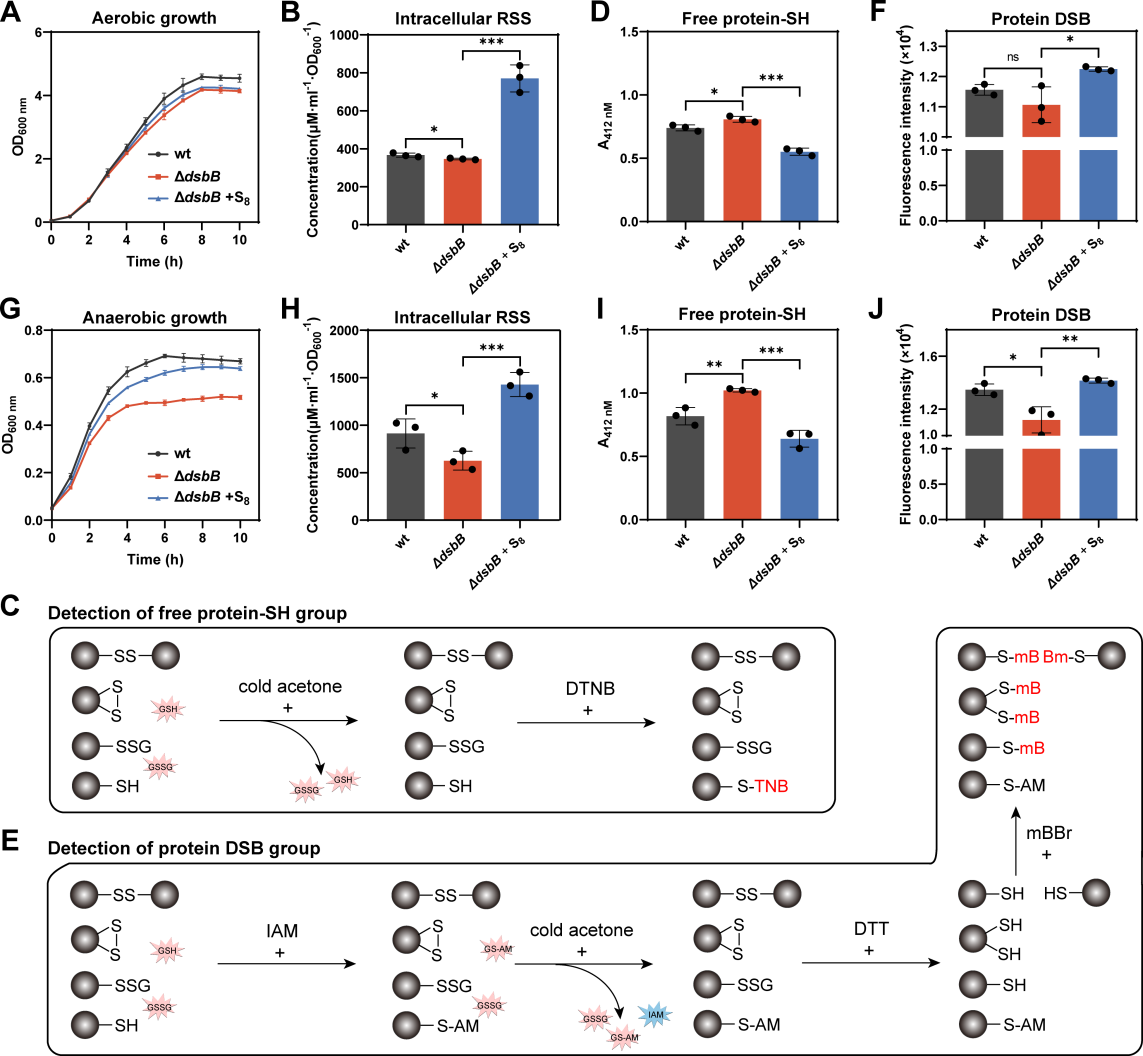
S_8_ complemented dsbB knockout under both aerobic and anaerobic conditions. (A) Growth of *E. coli* MG1655 wt, ΔdsbB, and S_8_-treated ΔdsbB under aerobic conditions. (B) Amounts of intracellular polysulfides in *E. coli* MG1655 strains under aerobic conditions. (C) Workflow of the DTNB based free protein–SH group quantification. (D) Amounts of the total free protein–SH group in *E. coli* MG1655 strains under aerobic conditions. (E) Workflow of the mBBr-based protein DSB group quantification. (F) Amounts of the total protein DSB group in *E. coli* MG1655 strains under aerobic conditions. (G) Growth of *E. coli* MG1655 strains under anaerobic conditions. (H) Amounts of intracellular polysulfides in *E. coli* MG1655 strain under anaerobic conditions. (I) Amounts of the total free protein–SH group in *E. coli* MG1655 strain under anaerobic conditions. (J) Amounts of the total protein DSB group in *E. coli* MG1655 strains under anaerobic conditions. Data were from three independent repeats and presented as mean value ±s.d. *P < 0.05, **P < 0.01, and ***P < 0.001 based on a two-sided t-test.

The DsbB protein serves as the oxidizing agent for DsbA, and together they mediate DSB formation in the periplasm of *E. coli*. We constructed a strain of *E. coli* MG1655 with *dsbB* deletion, termed Δ*dsbB*, and firstly cultivated it in LB medium under aerobic conditions. Compared with the wild-type *E. coli* MG1655 strain (wt), the Δ*dsbB* strain exhibited a subtle yet significant growth impairment (Fig. 4 and 8 ), indicating that disrupting the DSB system leads to growth defects. A previous study reported that S_8_ can enter cells by dissolving in the lipid bilayer of the cell membrane (30). We added 0.2 mM S_8_ into the Δ*dsbB* cultivation medium and found that, interestingly, the growth of Δ*dsbB* was partially recovered.

To further confirm the effect of S_8_, we collected 50 mL *E. coli* cells (OD_600_ = 1, cultivated under aerobic conditions) and resuspended them in 25 mL PBS buffer with or without 0.5 mM S_8_. After an incubation at room temperature for 30 min, cells were collected and subjected to further analysis. Without S_8_ treatment, the Δ*dsbB* strain contained slightly fewer intracellular polysulfides than the wt strain. When Δ*dsbB* strain was treated with S_8_, its intracellular polysulfides significantly increased and became about twofold higher than that of wt (Fig. 4B). Subsequently, the intracellular total free protein thiol (–SH) groups were quantified. Cells were harvested and then lysed using a high-pressure homogenizer. Their proteomes were isolated from low-molecular-weight molecules (including GSH and its oxidized form GSSG) using cold acetone precipitation (Fig. 4C). A probe that selectively tags free –SH groups, 5,5′-dithiobis (2-nitrobenzoic acid) (DTNB), was used to label the proteomes. After reaction with DTNB, the absorbance at 412 nm was measured. The Δ*dsbB* proteome displayed a higher absorbance value than did the wt proteome, indicating that the portion of proteins in -SH status was higher in Δ*dsbB* than that in wt (Fig. 4D). When Δ*dsbB* was treated with S_8_, the absorbance of its proteome became lower than that of wt proteome, suggesting that S_8_ decreased the portion of proteins in -SH status.

We then quantified the intracellular total DSB groups of the cells. After cell disruption, free protein–SH groups were blocked with iodoacetamide (IAM), and the proteomes were again isolated using cold acetone precipitation. The proteomes were then treated with DTT to convert their DSBs into free protein–SH groups. The resulting DSB-derived protein–SH groups were labeled with monobromobimane (mBBr) and quantified by measuring fluorescence intensity (Fig. 4E). There was no significant difference in fluorescence intensity between the Δ*dsbB* and wt proteomes (Fig. 4F). However, when Δ*dsbB* was treated with S_8_, the fluorescence intensity of its proteome significantly increased, suggesting that S_8_ increased the portion of proteins in DSB status.

Second, we cultivated Δ*dsbB* in LB medium under anaerobic conditions and examined its growth again. Different from results obtained from aerobic conditions, the Δ*dsbB* strain showed significantly impaired growth compared with wt strain under anaerobic conditions (Fig. 4G), but the growth was significantly recovered by adding 0.2 mM S_8_ into the cultivation medium. These observations suggest that polysulfides are more important for *E. coli* growth under anaerobic conditions than under aerobic conditions.

Furthermore, we collected 100 mL *E. coli* cells (OD_600_ = 0.5, cultivated under anaerobic conditions) and resuspended them in 25 mL PBS buffer (deoxygenated by purging with nitrogen for 30 min) with or without 0.5 mM S_8_. After incubation at room temperature for 30 min under anaerobic conditions, cells were collected and subjected to further analysis. When Δ*dsbB* was treated with S_8_, its intracellular polysulfides increased about threefold (Fig. 4H). The Δ*dsbB* strain had a substantially higher concentration of free protein–SH groups and a significantly lower concentration of protein DSB groups than the wt strain (Fig. 4I and J). S_8_ treatment significantly decreased the amount of free protein–SH groups and increased the protein DSB amount of Δ*dsbB* proteome.

Considering that LB is not the optimal medium for *E. coli* growth under anaerobic conditions, we chose a well-defined composition-M9 medium (M9 salt was supplemented with glucose and potassium nitrate) to perform the anaerobic experiments again. Under anaerobic conditions, Δ*dsbB* displayed severely impaired growth compared with wt (Fig. S7A), and S_8_ supplementation (0.2 mM) significantly restored its growth. Analysis of intracellular DSB groups showed a similar trend: DSB content was reduced in Δ*dsbB* but elevated after S_8_ treatment (Fig. S7B). These findings reinforce that polysulfides are particularly important for *E. coli* under anaerobic conditions.

In summary, these findings underscore the critical role of DsbB in DSB formation, but in the absence of DsbB, treating cells with polysulfides can compensate for its function, with the compensatory effect being particularly pronounced under anaerobic conditions.

### Polysulfides compensated for *dsbA* deletion in *E. coli*

**Fig 5:**
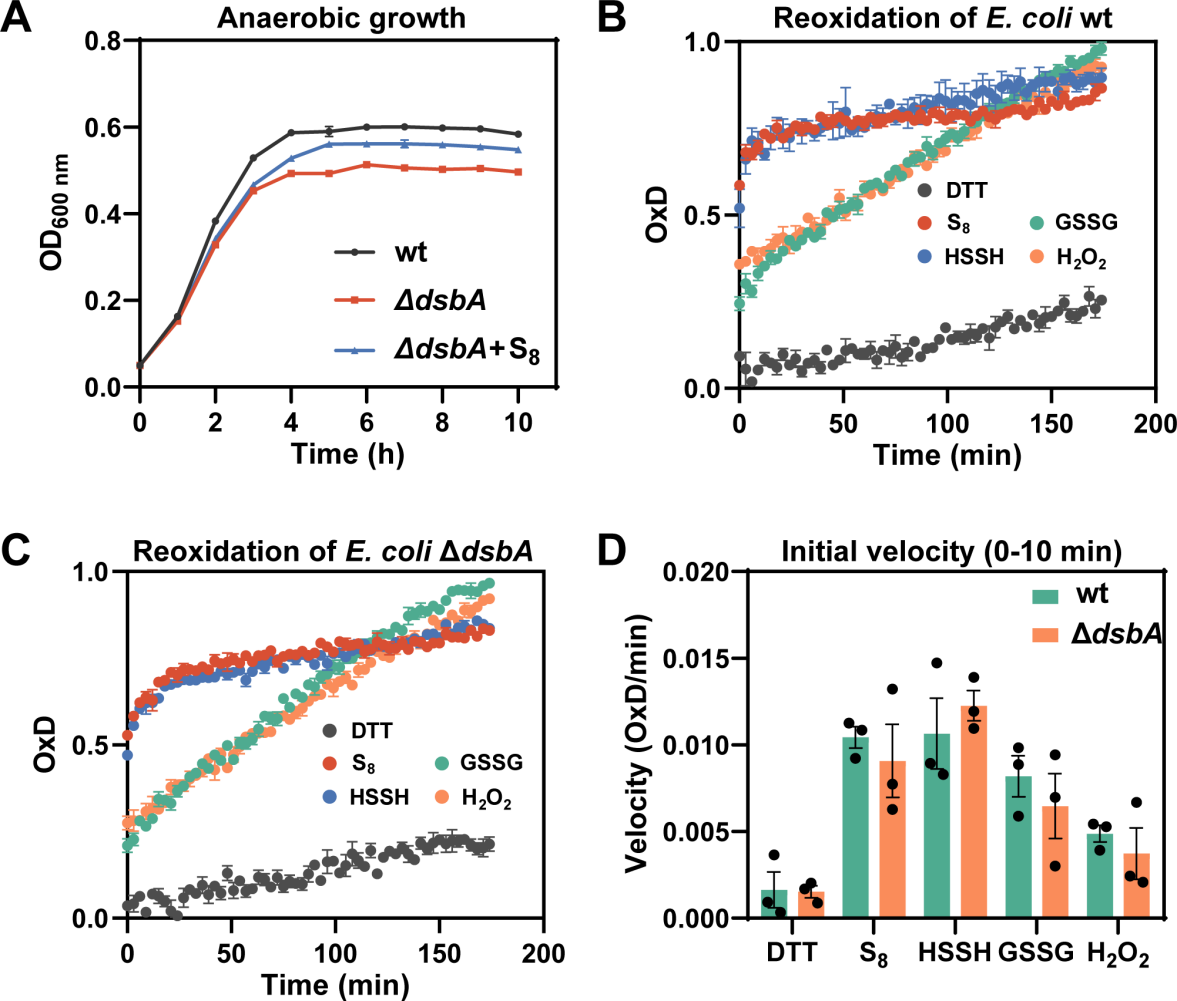
Polysulfides compensated periplasmic roGFP2 re-oxidation velocity in *E. coli* lacking DsbA. (**A**) Growth of *E. coli* MG1655 wt, Δ*dsbA*, and S_8_-treated Δ*dsbA* under anaerobic conditions. (**B, C**) Effect of various sulfur-containing compounds on the reoxidative capacity of roGFP2 in the periplasmic space of *E. coli. E. coli* was reduced with 10 mM DTT, washed to remove excess DTT, and treated with 0.5 mM S_8_, HSSH, GSSG, or H_2_O_2_. DTT-reduced cells served as a control. The fluorescence changes of roGFP2 were monitored over 180 min. (**D**) The initial reoxidation rate of roGFP2 in *E. coli* wt and Δ*dsbA* was measured during the first 10 min of the assay. Data were from three independent repeats and presented as mean value ±s.d.

DsbA directly reacts with target proteins to promote the formation of their DSBs. We also constructed a strain of *E. coli* MG1655 with *dsbA* deletion, termed Δ*dsbA*. As Δ*dsbB*, Δ*dsbA* showed also significant growth impairment in LB and composition-M9 medium under anaerobic conditions, and S_8_ treatment obviously recovered the growth (Fig. 5A; Fig. S8). To visualize the influence of *dsbA* deletion on DSB formation in the periplasm, we expressed the roGFP2 protein and located it in the periplasm of both wt and Δ*dsbA* strains. The strains were then treated with 0.5 mM S_8_, HSSH, GSSG, DTT, or H_2_O_2_ under anaerobic conditions. We observed that the ratio of oxidized roGFP2 (OxD) was quickly increased by S_8_ and HSSH treatment in both wt and Δ*dsbA* strains (Fig. 5B and C). GSSG and H_2_O_2_ also increased OxD, but their increasing rates were slower than those of S_8_ and HSSH. About the reoxidation rate calculated from the first 10 min, no obvious difference was observed between wt and Δ*dsbA* (Fig. 5D). These results suggest that polysulfides can promote periplasmic DSB formation independent of DsbA, and the promoting effects were more efficient than GSSG or H_2_O_2_.

### GSSH mediated protein S-glutathionylation

**Fig 6:**
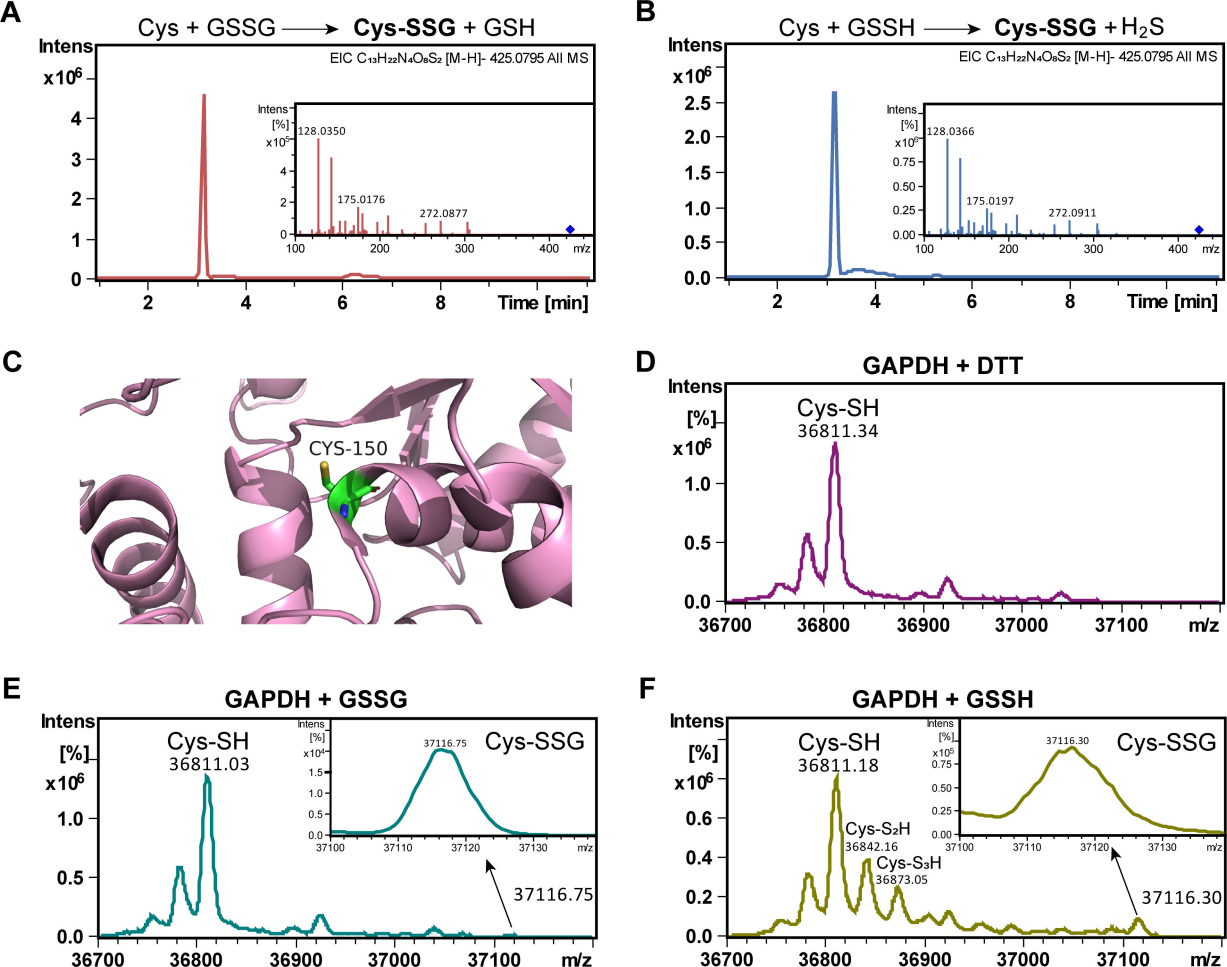
GSSH mediated S-glutathionylation formation in GAPDH. (**A**) LC–ESI-MS analysis of products from GSSG (5 mM) and cysteine (5 mM) reaction. (**B**) LC–ESI-MS analysis of products from GSSH (5 mM) and cysteine (5 mM) reaction. (**C**) 3D structure of GAPDH with Cys_150_ (PDB ID, 3PYM). (**D**) Full protein mass spectra of DTT-reacted GAPDH. (**E**) Full protein mass spectra of GSSG-reacted GAPDH. (**F**) Full protein mass spectra of GSSH-reacted GAPDH.

To assess the ability of GSSH to facilitate protein S-glutathionylation, which is the inter-DSB between proteins and GSH, we first mixed GSSH with cysteine at a 1:1 molar ratio under anaerobic conditions. The reaction proceeded at room temperature for 30 min, and the products were subsequently analyzed using LC–ESI-MS. For comparison, we also performed a reaction using GSSG as a positive control under identical conditions, as GSSG is known to mediate protein S-glutathionylation. The product cysteine-SSG was detected in both GSSH and GSSG reaction mixtures, with the MS signal intensities being 2.6 × 10^6^ for GSSH and 4.6 × 10^6^ for GSSG (Fig. 6A and B). These results indicate that both GSSH and GSSG can react with –SH group to form S-glutathionylation.

*S. cerevisiae* glyceraldehyde 3-phosphate dehydrogenase (GAPDH) is known to undergo S-glutathionylation in response to oxidative stress (Fig. 6C) (31). We procured purified GAPDH and mixed it with an excess of DTT, GSSG, or GSSH under anaerobic conditions. After 1 h incubation at room temperature, the modified GAPDH was analyzed by LC–MS. The DTT treated GAPDH displayed a principal molecular weight peak of 36,811, which matched its calculated molecular weight of the reduced form (Fig. 6D). For GSSG and GSSH-treated GAPDH, a minor molecular weight peak of 37,116 appeared, which corresponded to the GAPDH–SSG complex (Fig. 6E and F). Notably, the signal intensity of GAPDH–SSG was approximately fourfold higher in GSSH-treated GAPDH sample than that in GSSG-treated sample. These findings indicate that, similar to GSSG, GSSH can also mediate GAPDH S-glutathionylation.

It is noteworthy that except for GAPDH–SSG, we also detected GAPDH–S_2_H and GAPDH–S_3_H from the GSSH-reacted sample, but these modifications were not detected from the GSSG-reacted sample, indicating that GSSH can also lead to protein polysulfidation modification while GSSG has no such activity.

### Octasulfur increased DSB amount in other microorganisms

**Fig 7:**
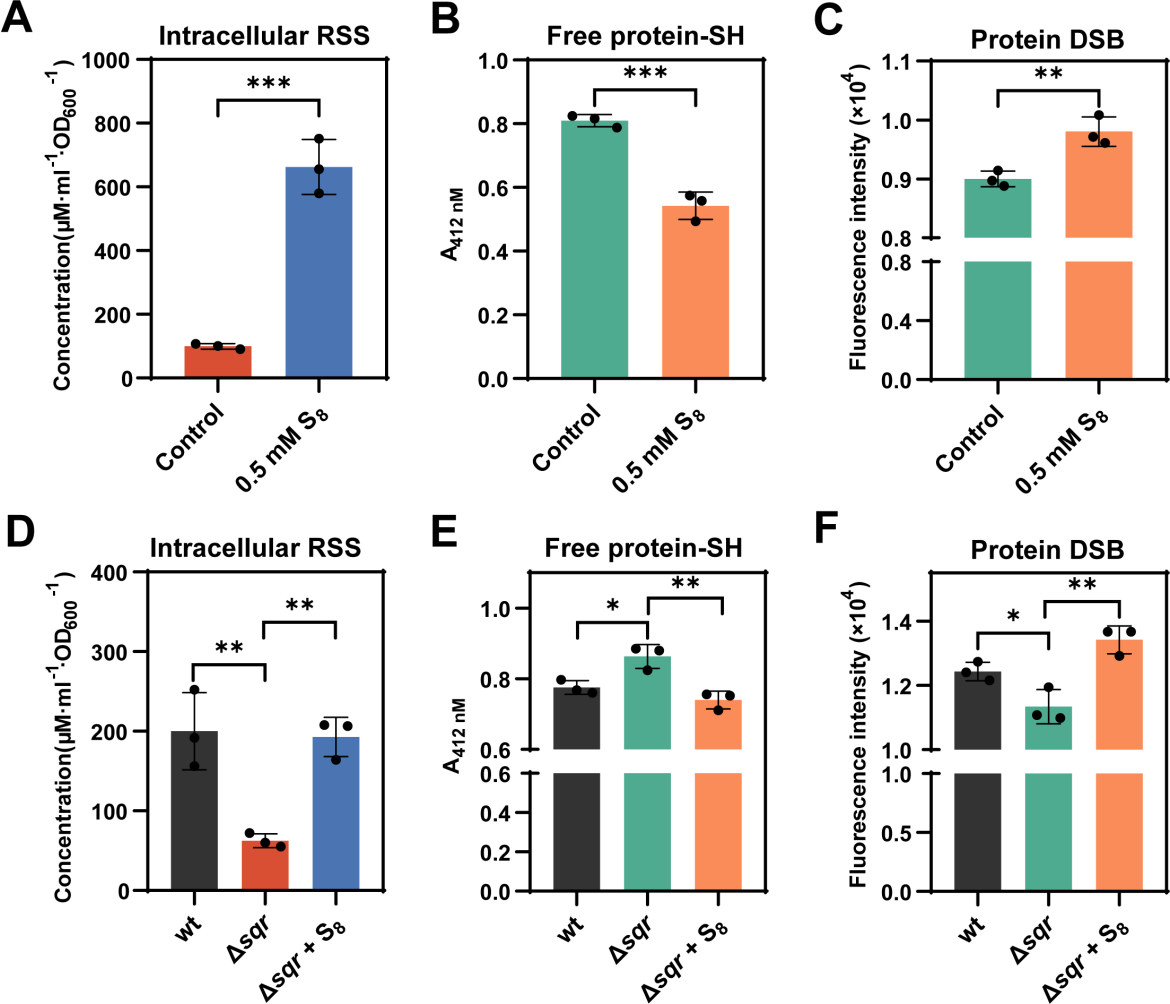
Correlation of intracellular polysulfides level with protein DSB level in *S. pombe* and *C. pinatubonensis* JMP134. (**A**) Treating *S. pombe* with S_8_ increased intracellular polysulfides levels. (**B**) Treating *S. pombe* with S_8_ caused a decrease in total free protein–SH group level. (**C**) Treating *S. pombe* with S_8_ caused an increase in total protein DSB group level. (**D**) Amounts of intracellular polysulfides in *C. pinatubonensis* JMP134 strains. (**E**) Amounts of the total free protein–SH group in *C. pinatubonensis* JMP134 strains. (**F**) Amounts of the total protein DSB group in *C. pinatubonensis* JMP134 strains under aerobic conditions. Data were from three independent repeats and presented as mean value ±s.d. *P < 0.05 and ***P < 0.001 based on a two-sided *t*-test. Data were from three independent repeats and presented as mean value ±s.d. *P < 0.05 and ***P < 0.001 based on a two-sided *t*-test.

To explore the influence of polysulfides on DSB formation of other microorganisms, we treated *S. pombe* cells (OD_600_ = 2) with 0.5 mM S_8_ under anaerobic conditions. Following a 30 min incubation at room temperature, the intracellular polysulfide content was measured. Exposure to S_8_ resulted in a substantial increase in intracellular polysulfides, approximately a sixfold enhancement relative to the untreated control group (Fig. 7A). Concurrently, there was a notable reduction in the free protein thiol (–SH) groups and a corresponding increase in protein DSB groups within the S_8_-treated cells (Fig. 7B and C).

Deletion of the sulfide:quinone oxidoreductase encoding gene (*sqr*) in *Cupriavidus pinatubonensis* JMP134 (Δ*sqr*) resulted in reduced intracellular polysulfide levels (Fig. 7D). We isolated proteomes from both wt and Δ*sqr* strains and examined the contents of free protein–SH and DSB groups. The proteome from Δ*sqr* strains contained a significantly higher concentration of free protein–SH groups and a lower concentration of DSB groups compared with wt cells. After treating the Δ*sqr* strain with S_8_, intracellular concentrations of the free protein–SH group and the DSB group were restored to levels similar to wt (Fig. 7E and F). These observations suggest that polysulfides also contribute to DSB formation in other microorganisms.

## DISCUSSION

In this study, we first verified that polysulfides can oxidize GSH to GSSG under anaerobic conditions. Building on this, we treated proteins, including roGFP2, Trx1, and DsbA, with polysulfides under anaerobic conditions and observed the formation of intra-molecular DSB in all three proteins. Further investigation involved the deletion of *dsbB* in *E. coli*. We observed a significant decrease in protein DSB levels in the mutant strain. Notably, treatment with S_8_ was able to reverse this decrease, with the most pronounced effects observed under anaerobic conditions. Treatment with S_8_ and HSSH promoted DSB formation of periplasmic roGFP2 quickly in *dsbA* deletion strain. Additionally, polysulfides are also associated with DSB formation in other microorganisms. Collectively, these results indicate that under anaerobic conditions, polysulfides can promote DSB formation independently of currently known DSB formation systems.

The recent work by Knoke et al. (32) reported that in DsbA-deficient *E. coli*, roGFP2 located in the periplasm can still form DSB, indicating the presence of an alternative system or player for DSB formation. They proposed that GSSG might directly oxidize periplasmic roGFP2 in DsbA-deficient *E. coli*. However, previous studies, including their own, showed that direct oxidation of roGFP2 by GSSG was inefficient. They speculated that an unidentified, GSH-dependent factor could oxidize roGFP2 in the absence of DsbA. Our previous studies have shown that intracellular polysulfide content was influenced by GSH levels and vice versa (33, 34). We have now demonstrated that polysulfides can directly oxidize roGFP2, providing an answer to their speculation—the unidentified factor is likely intracellular polysulfides.

The redox potentials of the *E. coli* periplasm and cytoplasm are approximately −160 and −260 mV, respectively (35, 36). DsbA, with a redox potential of −120 mV, is among the most oxidizing thiol–disulfide oxidoreductases (37), enabling it to maintain an oxidized state and mediate protein DSB formation in the periplasm. The standard redox potential of sulfane sulfur (S^0^ + 2H^+^ + 2e^−^ → H_2_S) is +144 mV, indicating that it can oxidize most proteins in both the periplasm and cytoplasm.

When S_8_ reacts with GSH, GSSH is produced as an intermediate, suggesting a two-step reaction mechanism (Reactions 1 and 2).


(1) $$GSH+S^{0}⟶GSSH$$

(2) $$GSSH+GSH⟶GSSG+H_{2}S$$

We hypothesize that polysulfides oxidize proteins through a similar mechanism. Initially, polysulfides transfer a sulfane sulfur atom to a cysteine residue on the target protein, forming a protein–SSH intermediate. Subsequently, the protein–SSH reacts with another cysteine to form a DSB, while the sulfane sulfur is reduced to H_2_S.

A previous report showed that GSSH can react with the glucose-induced biofilm accessory protein A (GbaA) to form GbaA-SSG (38). Herein, we found that GSSH can also react with GAPDH to form GAPDH-SSG. This activity of GSSH should be ascribed to its thiol-oxidizing activity conferred by the sulfane sulfur atom. The mechanism for protein S-glutathionylation by GSSH likely follows the same mechanism as reaction (2). In addition to DSB and S-glutathionylation, polysulfides can mediate a third modification, protein polysulfidation (protein–S_n_H, *n* ≥ 2). Actually, when using GSSH to react with GAPDH, we observed the generation of GAPDH–S_2_H and GAPDH–S_3_H (Fig. 5F), which were not observed from GSSG-reacted GAPDH. Therefore, the intracellular total free protein thiol groups (–SH) quantified with our method should contain a portion of the protein–S_n_H groups. However, due to the current lack of reliable protein–S_n_H quantification method, their contents cannot be determined so far (39, 40). On the other hand, the intracellular DSB groups quantified with our method may be modestly affected by the protein–S_n_H groups because a recent report indicated that protein–S_n_H groups labeled with IAM (protein–S_n_-AM) were not stable and easily converted to protein–S-AM (41), which should not disturb DSB quantification.

It is important to note that treating *dsbA* deletion strain with polysulfides can quickly oxidize its periplasmic roGFP2. For *dsbB* deletion strain, the compensatory effect of S_8_ also was more pronounced under anaerobic conditions compared with aerobic conditions. This correlates with the observation that polysulfides commonly accumulate in anaerobic habitats, such as coastal sediments and mammalian intestine (3, 42–44), where the DsbAB system cannot work efficiently and oxygen-related species are scarce. Historically, polysulfides predated oxygen and ROS. In the early stages of life, polysulfides may have already been mediating DSB formation. With the advent of the oxygen-rich era, this function persisted, becoming necessary primarily under anaerobic conditions.

## MATERIALS AND METHODS

### Strains and materials

All strains and plasmids are listed in Table S1. *E. coli* strains were grown in lysogeny broth (LB) medium or composition-M9 medium (M9 salts supplemented with glucose and potassium nitrate) at a temperature of 37°C with shaking (200 rpm). The construction of the *dsbB* knockout strain was carried out according to a previously published method (45). *S. pombe* HL6381 strains, characterized by the genotype (*h^+^ his3-D1 leu1-32 ura4-D18 ade6-M210*), were cultivated in yeast extract medium (YES) at 30°C with shaking (200 rpm). *C. pinatubonensis* JMP134 strains were cultivated in LB medium at a temperature of 30°C with shaking (200 rpm) (46).

GSH, GSSG, S_8_ (99.9% purity), sodium hydrosulfide (NaHS), diamide, cysteine, DTNB, and DTT were procured from Sigma-Aldrich (Shanghai, China). S_8_ solution was prepared as reported previously (47). Briefly, a saturation solution was made by dissolving excess sulfur powder in acetone. The concentration of saturated acetone sulfur is determined as 17 mM. HSSH was prepared following the established protocol (48). GSSH was synthesized using the method outlined in reference 49.

### Chemical reactions of polysulfides with thiols

For the reaction of S_8_ with GSH, reacting solutions used were purged with nitrogen for 30 min to remove dissolved oxygen, and then were placed in the anaerobic incubator. The mixing and incubating processes were conducted in the anaerobic incubator. GSH (5 mM) was prepared in KPI buffer (100 mM, pH 7.4). S_8_ solution was prepared by dissolving S_8_ powder in methanol in sealed bottle. GSH solution was mixed with S_8_ (5 mM) at room temperature. For the reactions of cysteine with GSSG or GSSH, the experiments also were conducted in the anaerobic incubator with nitrogen purged solutions. GSSG and GSSH solutions were prepared in KPI buffer (100 mM, pH 7.4). Cysteine (5 mM) was mixed with GSSG and GSSH solutions (5 mM) in an anaerobic incubator. After 30 min incubation at room temperature, the reaction was finished and the products were subjected to analysis.

For analysis, the reacted mixtures were subjected to centrifugation at 12,000*×g* for 3 min. The resulting supernatant was subsequently analyzed using liquid chromatography–electrospray ionization mass spectrometry (LC–ESI-MS).

### LC–ESI-MS analysis

The analysis procedure was performed as previously described (40). In summary, the samples underwent liquid chromatography (LC) using an InertSustain C18 column (Shimadzu, Japan) interfaced with a High-Resolution Q-TOF mass spectrometer (Ultimate 3000, Bruker impact HD, Germany). The samples were introduced into the LC system via the loading pump, with 0.25% acetic acid serving as the mobile phase A and 100% methanol as the mobile phase B. Over the initial minute, the concentration of mobile phase B was ramped up from 7.5% to 52.5% and held constant for 15 min. At the 15 min mark, the concentration of mobile phase B was further increased to 55%, then to 100%, and sustained for an additional 5 min. At 20.1 min, mobile phase B was reduced to 7.5% and maintained at this level until completion of the analysis at 31 min. The electrospray ionization (ESI) source temperature was set to 200°C, and the ion spray voltage was set at 4.5 kV. Nitrogen was employed as the nebulizer and drying gas. The acquired data were processed using Data Analysis 4.2 software.

### Protein expression and purification

*E. coli* BL21(DE3) strains containing the expression protein synthesis plasmids pET30-roGFP2, pET30-Trx1, pET30-DsbA, or pET30-Gapdh were cultivated in LB medium supplemented with 100 µg/mL kanamycin. Upon reaching an optical density (OD_600_) of 0.6–0.8, isopropyl β-D-1-thiogalactopyranoside (IPTG) was introduced to a final concentration of 0.3 mM to induce protein expression, and the culture was continued for an additional 18 h at 20°C. The bacterial cells were harvested by centrifugation and then re-suspended in a lysis buffer (50 mM NaH_2_PO_4_, 300 mM NaCl, 20 mM imidazole, 0.5 mM DTT, pH 8.0). Cell lysis was achieved using a high-pressure homogenizer, model SPCH-18 (Stansted), and the lysate was clarified by centrifugation at 12,000×*g* for 15 min. The supernatant was subsequently applied to a Ni-NTA agarose affinity resin for protein purification, following the manufacturer’s protocol. The purified protein was desalted using a PD-10 desalting column (GE Healthcare) that had been pre-equilibrated with a desalination buffer (0.5 mM DTT, 50 mM Tris-HCl, 10% glycerin, pH 7.4). The finally obtained protein solutions were stored in icebox in the anaerobic incubator before reacting with HSSH and other reagents. The purity of the protein was assessed by sodium dodecyl sulfate-polyacrylamide gel electrophoresis (SDS–PAGE), and protein concentration was quantified using a BCA protein assay kit (Beyotime Biotechnology, China).

### Polysulfides and ROS reactions with proteins

The roGFP2 was reacted with HSSH, S_8_, diamide, H_2_O_2_, dissolved O_2_, and DTT under aerobic conditions. The dissolved oxygen concentration was measured using a dissolved oxygen (DO) probe. The purified roGFP2 was diluted to a concentration of 0.3 mg/mL (10 µM) in KPI buffer (10 mM, pH 7.4). Subsequently, 100 µM of HSSH, H_2_O_2_, or DTT was introduced to the roGFP2 solution. The mixtures were incubated at room temperature for 30 min.

For the reaction of roGFP2 with HSSH under anaerobic conditions, reacting solutions used were purged with nitrogen for 30 min to remove dissolved oxygen. The mixing, incubating, and desalting processes were all conducted in the anaerobic incubator. The roGFP2 solution (10 µM) was mixed with 100 µM HSSH in KPI buffer (10 mM, pH 7.4) and incubated at room temperature for 30 min. Following the incubation period, the mixtures were processed through a PD-10 desalting column to eliminate any unreacted low-molecular-weight compounds. The roGFP2 samples post-reaction were then prepared for fluorescence analysis. The excitation spectra of roGFP2 were recorded using an RF-5301 PC fluorescence spectrophotometer (Shimadzu, Japan). The fluorescence intensities emitted at 511 nm (*E_m_* = 511 nm), when excited at 405 nm (*E_x_* = 405 nm) and 488 nm (*E_x_* = 488 nm), were quantified using a Synergy H1 microplate reader (BioTek, USA). The ratio of the emitted light intensities at these two excitation wavelengths (405/488) was calculated to assess the redox state of roGFP2.

For the reaction of Trx1 and DsbA with HSSH under anaerobic conditions, the reacting solutions used were purged with nitrogen for 30 min to remove dissolved oxygen. The mixing, incubating, and desalting processes were all conducted in the anaerobic incubator. Trx1 and DsbA were prepared at a concentration of 1.8 mg/mL (150 µM) and 3.2 mg/mL (150 µM), respectively. These were diluted in KPI buffer (10 mM, pH 7.4). Subsequently, 1.5 mM of HSSH was introduced into the mixture, which was then incubated under anaerobic conditions at room temperature for 30 min. Following the incubation period, the mixture underwent purification through a PD-10 desalting column to eliminate any unreacted low-molecular-weight reagents. The obtained protein samples were subsequently analyzed using LC–MS and LC–MS/MS.

For the reactions of GAPDH with GSSG and GSSH under anaerobic conditions, the reacting solutions used were purged with nitrogen for 30 min to remove dissolved oxygen. The mixing, incubating, and desalting processes were all conducted in the anaerobic incubator. GAPDH was diluted to 1.1 mg/mL (30 µM) in KPI buffer (10 mM, pH 7.4), 300 µM of GSSG or GSSH was added, and the mixture was incubated at room temperature for 1 h. After the incubation, the mixture was passed through a PD-10 desalting column to remove unreacted low-molecular-weight reagents. The obtained proteins were subjected to LC–MS analysis.

### Protein LC–MS and LC–MS/MS analysis

The molecular weight of the whole protein was analyzed using LC–MS. LC system equipped with an XBridge Protein BEH C4 Sentry Guard Cartridge (Waters, USA) and High-Resolution Q-TOF mass spectrometry (Ultimate 3000, Burker impact HD, GER) were used. The mobile phase A consisted of 0.1% formic acid (FA), while mobile phase B was a mixture of acetonitrile and 0.1% formic acid (FA). Initially, the concentration of mobile phase B was set at 5% for the first 7 min of the analysis. At the 7 min mark, the mobile phase composition was dynamically adjusted to a blend comprising 10% of 0.1% FA and 90% of the acetonitrile/0.1% FA mixture. This mixture was maintained for a duration of 3 min, after which mobile phase B was reduced back to 5%, and this conditions was sustained for an additional 3 min. The flow rate throughout the process was meticulously controlled at 0.5 mL/min. The Q-TOF mass spectrometer was equipped with an electrospray ionization (ESI) source operating in positive ion mode, with a capillary voltage set to 3500 V. The acquired data were subjected to deconvolution and comprehensive analysis using the Data Analysis 4.2 software.

For LC–MS/MS analysis, the protein sample was treated with HPE-IAM and subsequently digested with trypsin (0.5 mg/mL). The generated peptides were desalted by using a C18 column, eluted in 70% acetonitrile and 0.1% trifluoroacetic acid, and freeze-dried. The final product was resuspended in HPLC-grade water. LC–MS/MS analysis was performed using the Prominence nano-LC system (Shimadzu, Shanghai, China) equipped with a custom-made silica column (75 µm × 15 cm) packed with 3 µm Reprosil-Pur 120 C18-AQ. The elution process involved a 100 min gradient ranging from 0% to 100% of solvent B (0.1% formic acid in 98% acetonitrile) at a flow rate of 300 nL/min. Solvent A was composed of 0.1% formic acid in 2% acetonitrile. The eluent was ionized and electrosprayed via the LTQ-Orbitrap Velos Pro CID mass spectrometer (Thermo Scientific, Shanghai, China), which operated in data-dependent acquisition mode using Xcalibur 2.2.0 software (Thermo Scientific). Full-scan MS spectra (ranging from 400 to 1,800 m/z) were detected in the Orbitrap with a resolution of 60,000 at 400 m/z.

### Growth analysis of *E. coli* with S_8_ treatment

*E. coli* strains were initially cultivated in LB broth or composition-M9 medium overnight to ensure exponential growth. Subsequently, these cultures were subcultured into fresh LB medium (OD_600_ = 0.05), either supplemented with or without S_8_ solution (the final concentration of S_8_ in the medium was 0.2 mM). The cultivation was conducted under both aerobic and anaerobic conditions. For the aerobic conditions, 300 mL-scale shake-flasks containing 100 mL LB and inoculated *E. coli* were placed in a 37°C incubator with shaking (200 rpm). For the anaerobic conditions, the sealed serum bottles (250 mL scale) containing 100 mL LB medium and inoculated *E. coli* were purged with nitrogen for 30 min to remove dissolved oxygen. Samples were taken out using a 1 mL-scale syringe. The incubation temperature was maintained at 37°C throughout the experiment. The growth curves were determined by measuring the optical density of OD_600_ at 1 h intervals over a period of 10 h.

### S_8_ treatment of *E. coli, S*. *pombe,* and *C. pinatubonensis* JMP134

*E. coli* strains were cultivated in 50 mL LB medium or compostion-M9 medium at 37°C with shaking (200 rpm) under aerobic or anaerobic conditions. For *E.coli* cultivated under aerobic conditions, 50 mL cells were collected when OD_600_ reached 1. Cells were diluted in 25 mL PBS buffer (10 mM, pH 7.4) to make their OD_600_ = 2, then 0.7 mL S_8_ solution was added (the final S_8_ concentration was 0.5 mM). After 30 min incubation at 37°C with shaking (200 rpm), cells were collected and subjected to intracellular polysulfides, protein free thiol groups, and protein disulfide bond analysis. For *E. coli* cultivated under anaerobic conditions, 100 mL cells were collected when OD_600_ reached 0.5. The following processes were performed in the anaerobic incubator: cells were diluted in 25 mL PBS buffer (10 mM, pH 7.4, purged with nitrogen for 30 min before using) to make its OD_600_ = 2, then 0.7 mL S_8_ solution was added (the final S_8_ concentration was 0.5 mM). After 30 min incubation at 37°C with shaking (200 rpm), cells were collected and resuspened in different reaction buffers suitable for intracellular polysulfides, protein free thiol groups, and protein disulfide bond analysis.

*S. pombe* HL6381 was cultivated in 50 mL YES medium at 30°C with shaking (200 rpm). When OD_600_ reached 1, cell cultures were collected. Cells were diluted in 25 mL PBS buffer (10 mM, pH 7.4) to make their OD_600_ = 2, then 0.7 mL S_8_ solution was added (the final S_8_ concentration was 0.5 mM). After 30 min incubation at 30°C with shaking (200 rpm), cells were collected and subjected to different reaction buffers suitable for intracellular polysulfides, protein free thiol groups, and protein disulfide bond analysis.

*C. pinatubonensis* JMP134 was cultivated in 50 mL LB medium at 30°C with shaking (200 rpm). When OD_600_ reached 1, cell cultures were collected. Cells were diluted in 25 mL PBS buffer (10 mM, pH 7.4) to make their OD_600_ = 2, then 0.7 mL S_8_ solution was added (the final S_8_ concentration was 0.5 mM). After 30 min incubation at 30°C with shaking (200 rpm), cells were collected and subjected to different reaction buffers suitable for intracellular polysulfides, protein free thiol groups, and protein disulfide bond analysis.

### Quantification of intracellular polysulfides in *E. coli*, *S. pombe,* and *C. pinatubonensis* JMP134

For *E. coli, S. pombe,* and *C. pinatubonensis* JMP134 cells, S_8_ treatment was performed as described above.

The intracellular levels of polysulfides were quantified as a method previously reported in the literature (50). Briefly, 1.5 mL S_8_ treated *E. coli*, *S. pombe*, and *Cp* JMP134 cells obtained from one culture dish were collected and resuspended in 100 μL reaction buffer (50 mM Tris-HCl, 1% Triton X-100, 50 mM sulfite, and 50 µM diethylene triamine pentaacetic acid, pH 9.5). This suspension was subjected to thermal treatment by incubating at 95°C for 10 min, a step designed to convert intracellular polysulfides into thiosulfate. Following the incubation, the reaction mixture was centrifuged to separate the components. A 50 µL aliquot of the supernatant was carefully pipetted out for further analysis. This supernatant was then incubated with 5 µL of mBBr solution at a concentration of 25 mM, at room temperature for 30 min to facilitate the derivatization of thiosulfate. To terminate the reaction, 55 µL of a mixture of acetic acid and acetonitrile (in a volume ratio of 1:9) was added. After the addition of the stopping solution, the mixture was centrifuged to collect the supernatant layer. The concentration of thiosulfate present in this supernatant was determined using high-performance liquid chromatography (HPLC) on a Shimadzu system from Japan.

### Detection of intracellular protein free thiol groups and protein disulfide bonds

For *E. coli, S. pombe,* and *C. pinatubonensis* JMP134, S_8_ treatment was performed as described above.

For detection of intracellular protein free thiol groups, 25 mL S_8_-treated *E. coli*, *S. pombe,* and *C. pinatubonensis* JMP134 cells (OD_600_ = 50) were collected and resuspended in 10 mL PBS buffer (10 mM, pH 7.4) supplemented with 1% protease inhibitors to prevent unwanted proteolysis. Subsequently, the cell suspensions were subjected to lysis using a high-pressure homogenizer, specifically the SPCH-18 model from Stansted. The lysed cells were then centrifuged at 12,000*×g* for 15 min to separate the soluble proteins in the supernatant. The supernatant was treated with cold acetone to precipitate the proteins. A volume of 500 µL of the supernatant was mixed with 1.5 mL of 100% pre-chilled acetone, and the mixture was incubated at −20°C for 90 min to facilitate protein precipitation. Following the incubation, the mixture was centrifuged again at 14,000*×g* for 10 min to pellet the proteins. The supernatant was carefully removed, and the acetone was allowed to evaporate at room temperature, yielding a protein pellet. The protein pellet was then resuspended in a denaturing buffer (8 M urea) to solubilize the proteins. The resulting protein solution was diluted to a concentration of 5 mg/mL. To this solution, 20 mM DTNB was added to initiate the reaction. This mixture was incubated at room temperature for 15 min. Finally, the light absorbance of the reacted proteins at a wavelength of 412 nm was measured using a Synergy H1 microplate reader from BioTek, USA.

The intracellular concentration of protein DSB was quantified as previously reported (51). Briefly, 25 mL S_8_-treated *E. coli* and *S. pombe* cells (OD_600_ = 50) were collected and resuspended in 10 mL PBS buffer solution (100 mM, pH 8.0) supplemented with 20 mM NaCl, 1 mM EDTA, 200 mM iodoacetamide (IAM), and 1% protease inhibitor. The cell suspensions were then subjected to lysis using the high-pressure homogenizer SPCH-18 from Stansted. Following lysis, the supernatant was harvested by centrifugation at 12,000*×g* for 15 min. A 500 µL aliquot of the supernatant was treated with IAM and incubated at 37°C for 1 h in a light-protected environment to alkylate and block any free thiol groups present in the proteins. To eliminate unreacted IAM, the protein solution was precipitated using a threefold volume of pre-chilled acetone and incubated at −20°C for 90 min. After this incubation, the mixture was centrifuged at 14,000*×g* for 10 min. The supernatant was carefully removed, and the acetone was allowed to evaporate at room temperature, yielding a protein pellet. The protein pellet was then resuspended in a denaturing buffer (8 M urea). The protein solution was diluted to a concentration of 1 mg/mL and mixed with 1 mM dithiothreitol (DTT). This mixture was incubated at 37°C for 30 min to reduce any disulfide bonds present in the proteins. Subsequently, the newly liberated thiol groups were reacted with 2.5 mM mBBr, and the reaction was allowed to proceed at room temperature for 20 min. The fluorescence resulting from the mBBr–thiol adduct was measured using a Synergy H1 microplate reader from BioTek, USA. The fluorescence intensities were recorded with excitation at 370 nm (*E_x_* = 370 nm) and emission at 485 nm (*E_m_* = 485 nm).

### Detection of roGFP2 reoxidation capacity in the periplasmic space of *E. coli*

To localize roGFP2 into the periplasmic space, plasmids were constructed following the method described in reference 52. PCR was performed to amplify the torA signal sequence using the MG1655 genome as a template. The torA signal sequence was fused with a sequence of roGFP2, and the resulting product was then ligated into the plasmid ptrc99a using In-Fusion assembly master mix (Takara, JP).

The reoxidative capacity of roGFP2 in *E. coli* wt and Δ*dsbA* strains was assayed based on the method described in reference 31. Plasmid ptrc99a-torA(SP)-roGFP2 was first transformed into *E. coli* wt and Δ*dsbA* strains. These strains were cultured in LB medium at 37°C and 200 rpm until the OD_600_ reached ~0.4–0.6. Then, 0.5 mM IPTG was added, and the cultures were incubated at 20°C for 16 h. The cells were collected and washed twice with HEPES buffer (40 mM, pH 7.4, purged with nitrogen for 30 min to remove dissolved oxygen), resuspended in the same buffer to adjust the OD_600_ to 1.0 under anaerobic conditions. The resuspended cells were mixed with 10 mM DTT or 1 mM H_2_O_2_ and incubated at room temperature for 30 min. To detect the reoxidative capacity of different reagents, cells were first treated with 10 mM DTT to reduce the periplasmic roGFP2, and then were washed with deoxygenated HEPES buffer to remove excess DTT. The reagents, including 0.5 mM S_8_, 0.5 mM HSSH, 0.5 mM GSSG, 0.5 mM H_2_O_2_, or 0.5 mM DTT, were then used to treat these cells under anaerobic conditions. After 30 min incubation at room temperature, 100 µL of the bacterial suspension was transferred to a wells of a black, clear-bottom 96-well plate, and fluorescence was detected using a Synergy H1 microplate reader (BioTek, USA) with emission at 515 nm and excitation at 405 and 488 nm, respectively. The oxidation state of the probe was calculated using the fluorescence intensity ratio at the excitation wavelengths of 405 and 488 nm. All values were normalized using the following equation:


$$OxD=\frac{R-R_{red}}{\left(\frac{I_{488ox}}{I_{488red}})*(R_{ox}-R)+(R-R_{red}\right)}$$

where *R*_ox_ and *R*_red_ are the 405 nm/488 nm ratios for fully oxidized (1 mM H_2_O_2_ treatment) and fully reduced (10 mM DTT treatment) roGFP2, respectively. *I*_488ox_ and *I*_488red_ are the fluorescence intensities of roGFP2 at 488 nm under oxidizing and reducing conditions, respectively. *R* is the 405 nm/488 nm ratio of roGFP2 in *E. coli* strains treated with different reagents.

### Statistical analysis

Statistical analysis was done using GraphPad Prism 9.0. Data are presented as mean ± s.d. The significant difference between the two groups was analyzed using an independent Student’s *t*-test; *P* < 0.05 indicated statistical significance.

## Data Availability

All data needed to evaluate the conclusions in the paper are present in the paper and/or the supplemental material. Additional data related to this paper may be requested from the authors.

## SUPPLEMENTAL MATERIAL

Supplemental material aem.01926-24-s0001.docx Figures S1 to S8; Tables S1 and S2.

ASM does not own the copyrights to Supplemental Material that may be linked to, or accessed through, an article. The authors have granted ASM a non-exclusive, world-wide license to publish the Supplemental Material files. Please contact the corresponding author directly for reuse.
