# Supplementary material for: Polysulfides promote protein disulfide bond formation in microorganisms growing under anaerobic conditions
Source: Appl Environ Microbiol. 2025 Feb 7;91(3):e01926-24. doi: 10.1128/aem.01926-24 (PMC11921322; doi:10.1128/aem.01926-24)
Supplement: Supplemental material — Figures S1 to S8; Tables S1 and S2. [file aem.01926-24-s0001.docx]

Supplementary Materials for

Polysulfides promote protein disulfide bond formation in microorganisms growing under anaerobic condition

Yuping Xin^1^, Qingda Wang^1^, Jianming Yang^2^, Xiaohua Wu^1^, Yongzhen Xia^1^, Luying Xun^1,3^, Huaiwei Liu^1^*

^1^State Key Laboratory of Microbial Technology, Shandong University, Qingdao, 266200, People’s Republic of China.

^2^College of Life Sciences, Qingdao Agricultural University, Qingdao, 266109, People’s Republic of China.

^3^School of Molecular Biosciences, Washington State University, Pullman, WA, 991647520, USA.

*Corresponding author: Huaiwei Liu. Email: liuhuaiwei@sdu.edu.cn

**This file includes:**

Figs. S1 to S8

Tables S1 to S2

Fig. S1.


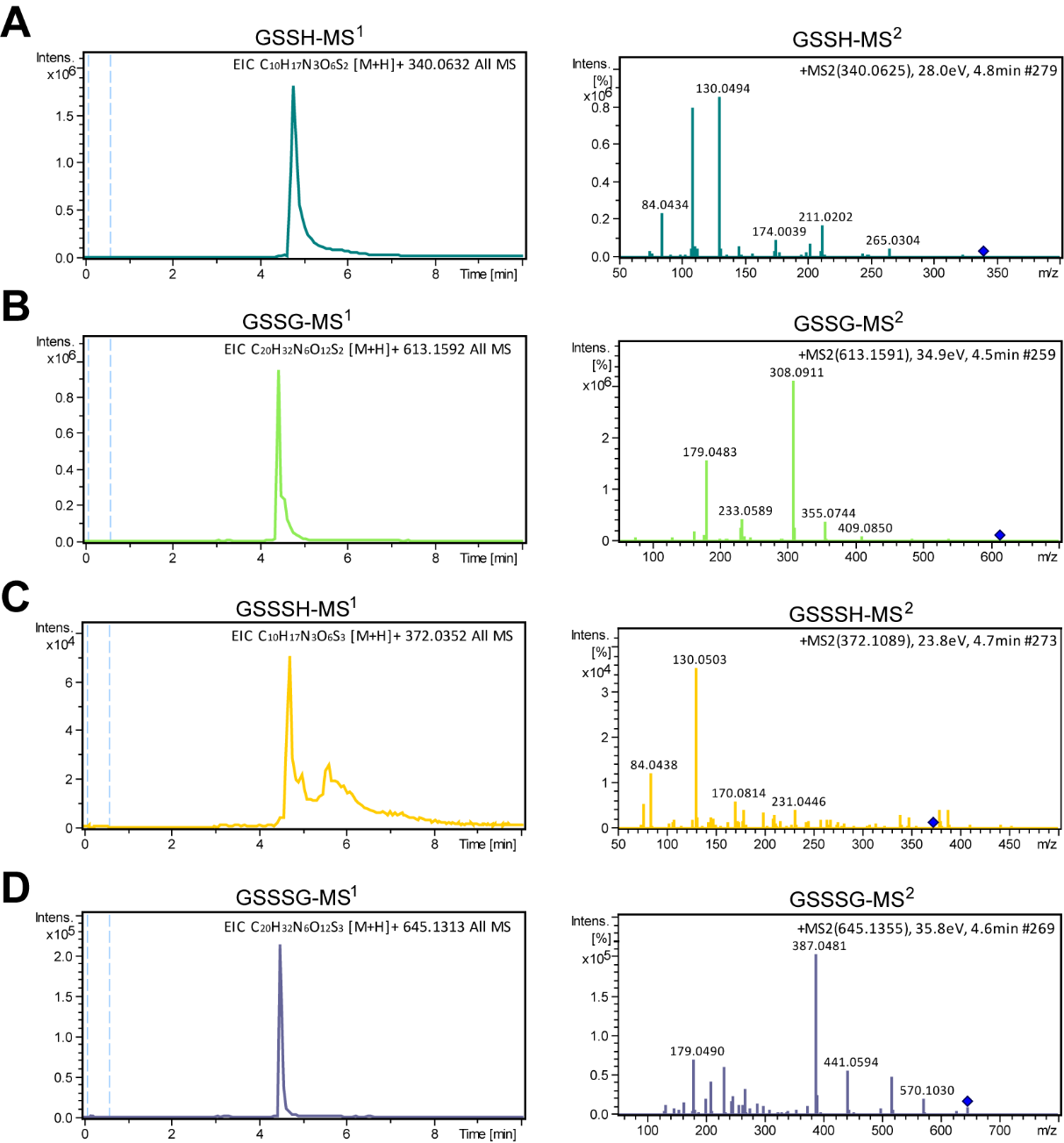


Fig. S1. Mass spectra of GSSH, GSSG, GSSSH, and GSSSG that were produced from S_8_ + GSH reaction. (A) MS^1^ and MS^2^ spectra of GSSH. (B) MS^1^ and MS^2^ spectra of GSSG. (C) MS^1^ and MS^2^ spectra of GSSSH. (D) MS^1^ and MS^2^ spectra of GSSSG.

**Fig. S2.**

**
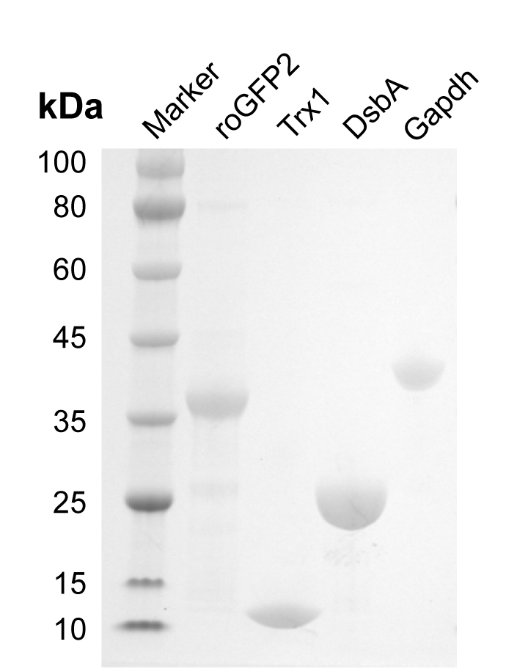
**

**Fig. S2. SDS-PAGE analysis of roGFP2, Trx1, DsbA, Gapdh.**

**Fig. S3.**


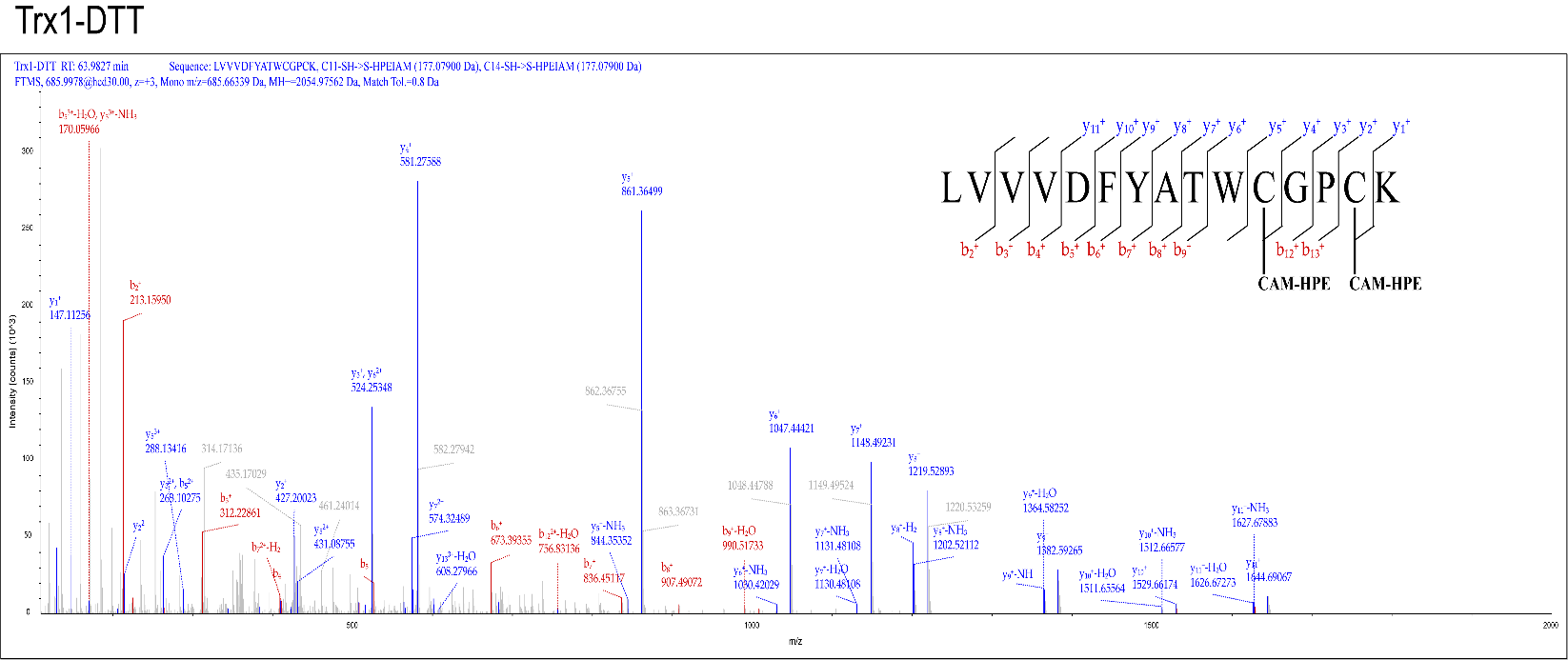


**Fig. S3. LC-MS/MS spectra showing that C_30_ and C_33_ in LVVVDFYATWCGPCK peptide of Trx1 were directly blocked by HPE-IAM.**

**Fig. S4.**


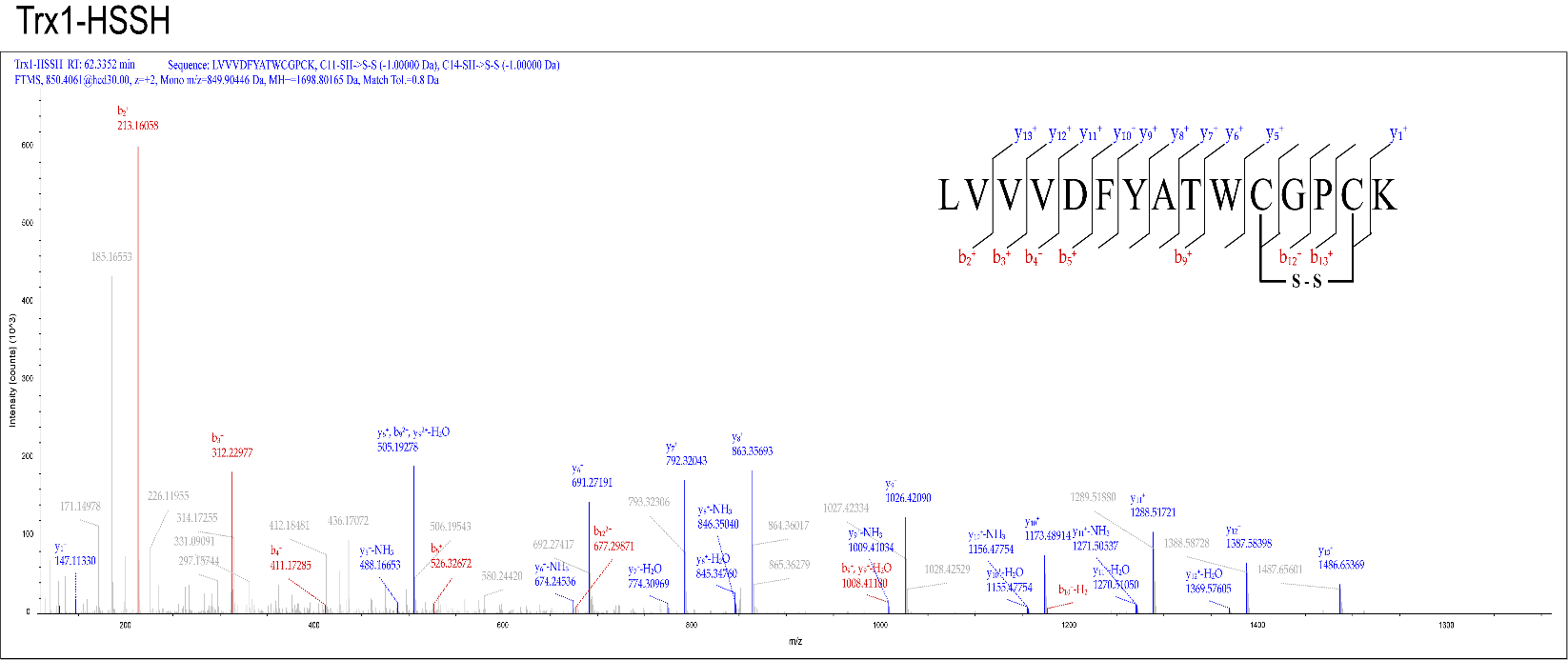


**Fig. S4. LC-MS/MS spectra showing that intramolecular disulfide bond formed between C_30_ and C_33_ in the LVVVDFYATWCGPCK peptide of Trx1.**

**Fig. S5.**


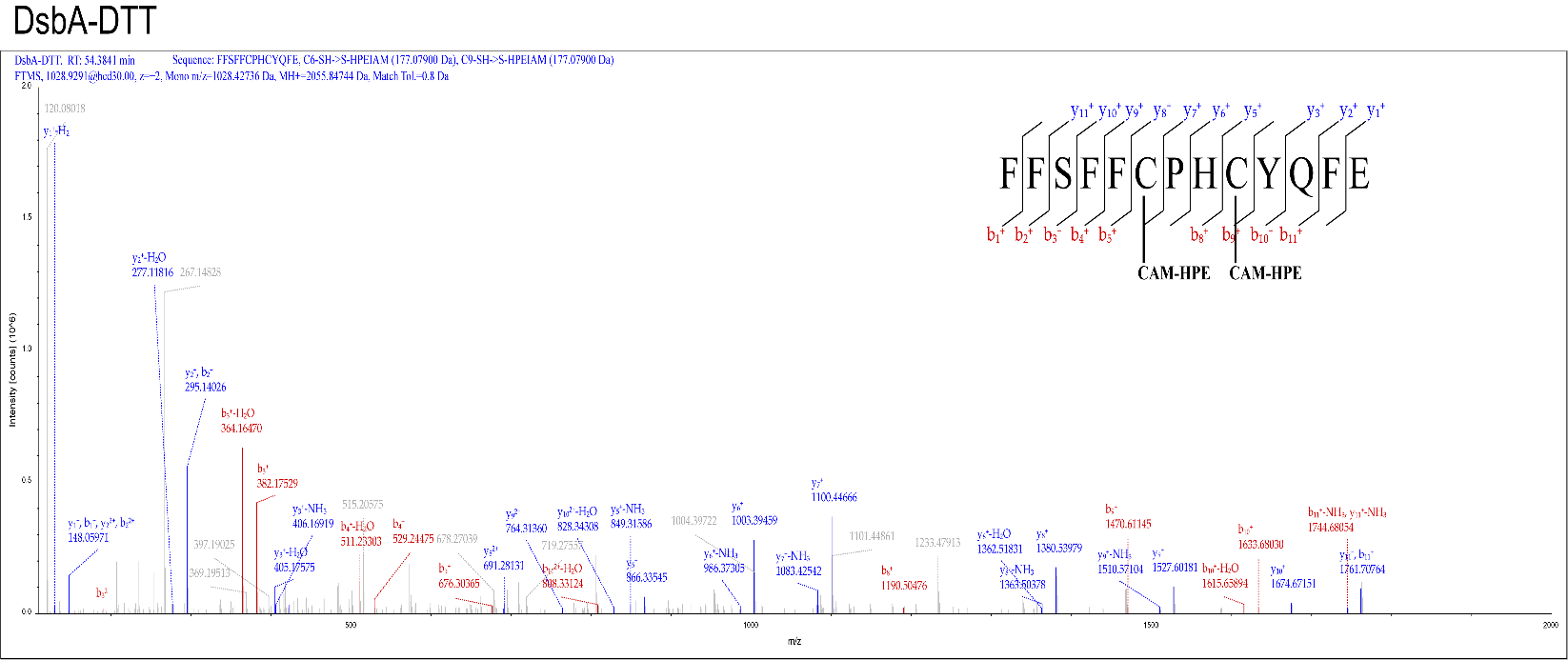


**Fig. S5. LC-MS/MS spectra showing that C_30_ and C_33_ in FFSFFCPHCYQFE peptide of DsbA was directly blocked by HPE-IAM.**

**Fig. S6.**


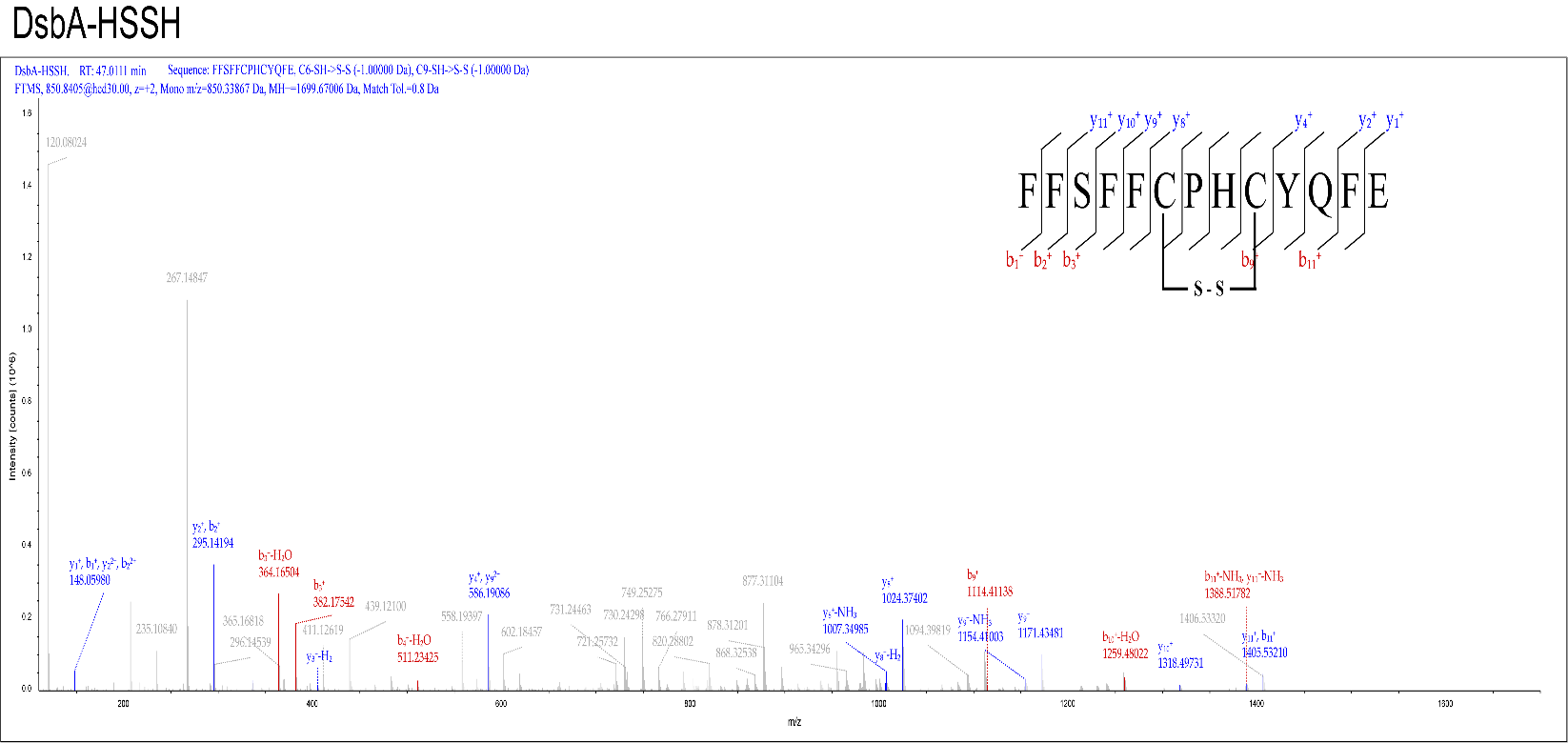


**Fig. S6. LC-MS/MS spectra showing that intramolecular disulfide bond formed between C_30_ and C_33_ in FFSFFCPHCYQFE peptide of DsbA.**

**
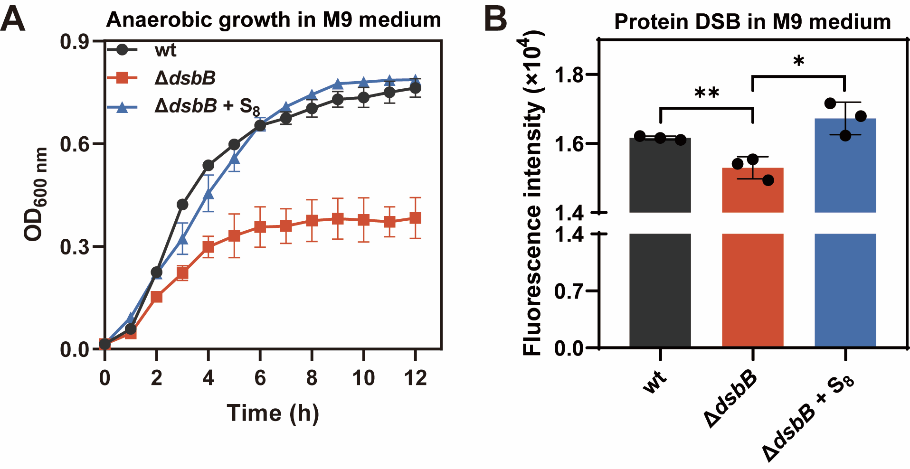
**

**Fig. S7. S_8_ complemented *dsbB* deletion under anaerobic conditions in composition M9 medium.** (A) Growth of *E. coli* MG1655 wt, Δ*dsbB*, and S_8_-treated Δ*dsbB* under anaerobic conditions in composition M9 medium. (B) Amounts of the total protein DSB group in *E. coli* MG1655 strains under anaerobic conditions in M9 medium.

**
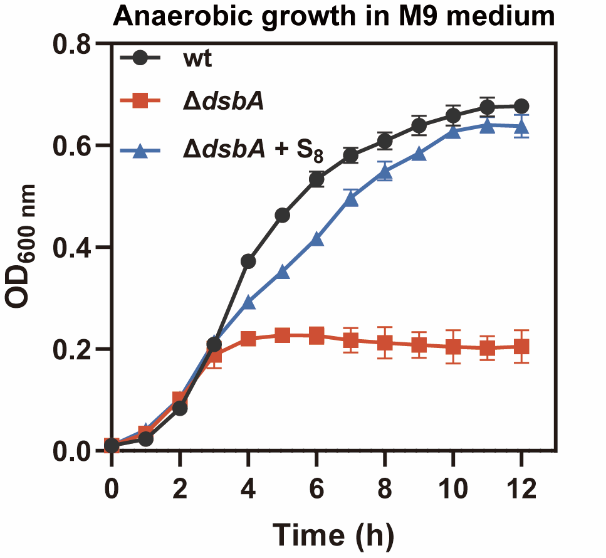
**

**Fig. S8.** **Growth of *E. coli* MG1655 wt, Δ*dsbA*, and S_8_-treated Δ*dsbA* under anaerobic conditions in composition M9 medium.**

Table S1. Strains and plasmids used in this study

| Strain/plasmid | Characteristic | Source |
| --- | --- | --- |
| ***Escherichia coli* strains** |  |  |
| MG1655 | *K-12 F– λ– ilvG– rfb-50 rph-1* | Lab stock |
| MG1655 Δ*dsbB* | *dsbB* deletion mutant of *E. coli* MG1655 | This study |
| MG1655 Δ*dsbA* | *dsbA* deletion mutant of *E. coli* MG1655 | This study |
| DH5α | *supE44 ΔlacU169(Φ80dlacZΔM15) hsdR17 recA1 endA1 gyrA96 thi-1 relA1* | Lab stock |
| BL21(DE3) | *F-ompT hsdSB (rB-mB-) gal (λ1857 ind1 Sam7 nin5 lacUV5 T7gene1) dcm.* | Lab stock |
| ***Schizosaccharomycs.pombe*** |  |  |
| yHL6381 | *h^+^ his3-D1 leu1-32 ura4-D18 ade6-M210* | Lab stock |
| ***Cupriavidus pinatubonensis***  JMP134 | Wild type | Lab stock  Lab stock |
| JMP134 Δ*sqr* | *sqr* deletion mutant of JMP134 |  |
| **Plasmids** |  |  |
| pET30 Ek/LIC | Expression plasmid in *E. coli* | Lab stock |
| pET30-roGFP2 | *roGFP2* in pET30, control by IPTG-induced lac promoter | Lab stock |
| pET30-trx1 | *trx1* in pET30, control by IPTG-induced lac promoter | This study |
| pET30-dsbA | *dsbA* in pET30, control by IPTG-induced lac promoter | This study |
| pET30-gapdh | *gapdh* in pET30, control by IPTG-induced lac promoter | This study |
| pTrc99a-tora(SP)-roGFP2 | Ampicillin resistance, trc promoter, used for expression of roGFP2 in periplasmic space | This study |
| pTKred | pSC101 ori, temperature sensitive, low copy, used for gene deletion | Lab stock |
| pKD4 | R6K ori, Kan^R^ and Amp^R^ used for gene deletion or template of pKat promoter | Lab stock |
| pCP20 | pSC101 ori, temperature sensitive, low copy, used for antibiotic resistance gene removing | Lab stock |

Table S2. Primers used in this study.

| Primers | Sequence (5'-3') | Description |
| --- | --- | --- |
| up-dsbB-F | gaattggtttaaactgcgcactctatgcatattgcagggaaatgattatgttgGATTGCAGCATTACACGTCTTG | Used for *dsbB* deletion |
| down-dsbB-R | gaaaaaagcgctcccgcaggagcgccgaatggattagcgaccgaacagatcGGGAATTAGCCATGGTCCATATG |  |
| YZ-dsbB-out-F | cttacaccctcgtcctgacac |  |
| YZ-dsbB-out-R | cactctattttgccgggcag |  |
| YZ-dsbB-in-F | ctgctctggcactggaact |  |
| YZ-dsbB-in-R | cttgcggcacccacttatc |  |
| up-dsbA-F | acggctttatgtaatttacattgaattattttttctcggacagatatttc TTGCAGCATTACACGTCTTGAG | Used for *dsbA* deletion |
| down-dsbA-R | ccctttgcaattaacacctatgtattaattcggagagagtagatcatgaaa GGGAATTAGCCATGGTCCATATG |  |
| YZ-dsbA-out-F | CGACAGACGGCGACTTTTATAG |  |
| YZ-dsbA-out-R | AGCGGCAGGATGCATTATCA |  |
| YZ-dsbA-in-F | GCGCAGTATGAAGATGGTAAACA |  |
| YZ-dsbA-in-R | ATTGCTGGTATCCATACCCTG |  |
| torA(SP)-F | atggccaataacgatctctttcaggcatcacgt | Used for amplification of torA signal sequences |
| torA(SP)-R | cttgcgccgcagtcgcac |  |
| roGFP2-F | gcgactgcggcgcaagcggcgGCTGATAGTAAAGGAGAAGAACTTTTCACT | Used for amplification of roGFP2 |
| roGFP2-R | CTATTTGTATAGTTCATCCATGCCATGTG |  |
| pTrc99a-F | GATGAACTATACAAATAGgagaagattttcagcctgatacagat | Used for amplification of pTrc99a |
| pTrc99a-R | aaagagatcgttattggccatggtctgtttcctgtgtgaaattgt |  |
| S.c-trx1-F | ctttaagaaggagatatacatATGGTTACTCAATTCAAAACTGCCAG | Used for pET30-Trx1 plasmid construction |
| S.c-trx1-R | gtggtggtggtggtgctcgagAGCATTAGCAGCAATGGCTTG |  |
| *E.coli*-dsba-F | ctttaagaaggagatatacatATGGCGCAGTATGAAGATGG | Used for pET30-DsbA plasmid construction |
| *E.coli*-dsba-R | gtggtggtggtggtgctcgagTTTTTTCTCGGACAGATATTTCACTGTATCAG |  |
| Tdh3-F | CTTTAAGAAGGAGATATACATATGGTTAGAGTTGCTATTAACGG | Used for pET30-Gapdh plasmid construction |
| Tdh3-R | GTGGTGGTGGTGGTGGTGCTCGAGAGCCTTGGCAACGTGTTCAAC |  |
